# 9.4‐Tesla magnetic resonance imaging in focal epilepsy patients with high‐resolution surface‐based profiling of focal cortical dysplasias

**DOI:** 10.1002/epi.70418

**Published:** 2026-09-02

**Authors:** Cornelius Kronlage, Pascal Martin, Benjamin Bender, Gisela E. Hagberg, Jonas Bause, Joana R. A. Loureiro, Mathilde Ripart, Sophie Adler, Konrad Wagstyl, Holger Lerche, Niels K. N. Focke, Esther Kuehn

**Affiliations:** ^1^ Department of Neurology and Epileptology University of Tübingen Medical Faculty Tübingen Germany; ^2^ Hertie Institute for Clinical Brain Research University of Tübingen Medical Faculty Tübingen Germany; ^3^ School of Biomedical Engineering and Imaging Sciences King's College London London UK; ^4^ Department of Neuroradiology University Hospital Tübingen and University of Tübingen Tübingen Germany; ^5^ Department of High‐Field Magnetic Resonance Max Planck Institute for Biological Cybernetics Tübingen Germany; ^6^ Biomedical Magnetic Resonance University Hospital of Tübingen Tübingen Germany; ^7^ UCL Great Ormond Street Institute of Child Health University College London London UK; ^8^ Department of Neurology University Medical Center Göttingen Göttingen Germany; ^9^ German Center for Neurodegenerative Diseases Tübingen Germany; ^10^ Present address: Siemens Healthineers Erlangen Germany

**Keywords:** black line sign, laynii, morphometry, MP2RAGE, quantitative, ultra‐high field

## Abstract

**Objective:**

The detection of subtle epileptogenic lesions such as focal cortical dysplasias (FCDs) is a clinical challenge in the management of drug‐resistant focal epilepsy (DRFE). Ultra‐high‐field (UHF) magnetic resonance imaging (MRI) offers increased signal‐to‐noise ratios and spatial resolution compared to 3‐T MRI and may improve diagnostic yield.

**Methods:**

We recruited *n* = 21 DRFE patients (with 3‐T MRI findings: two positive, three equivocal, 16 negative) undergoing presurgical workup and *n* = 20 healthy controls for 9.4‐T MRI (.8 mm isotropic magnetization‐prepared 2 rapid acquisition gradient echo [MP2RAGE], slabs of .375 × .375 × .8 mm T2*‐weighted gradient echo) and 3‐T MRI (magnetization prepared rapid acquisition gradient echo [MPRAGE], magnetization‐prepared 2 rapid acquisition gradient echo [MP2RAGE], fluid‐attenuated inversion recovery [FLAIR]) acquisitions. Visual review for possible epileptogenic lesions was performed by clinical experts. For histopathologically confirmed FCDs, we extracted surface‐based quantitative features (cortical thickness, quantitative T1, FLAIR, T2*, and quantitative susceptibility mapping values) across cortical depths and distances from the lesion center and performed high‐resolution cortical profiling of 9.4‐T T2* values.

**Results:**

In two patients with histopathologically confirmed FCD IIb, lesions were visible with distinct qualitative and quantitative features at both field strengths. One of these type IIb FCDs showed a focal cortical T2* reduction at 9.4 T that could be quantified via automated cortical profiling, consistent with the previously described “black line sign.” No new epileptogenic lesions were identified at 9.4 T in 3‐T MRI‐negative patients, who also had no histological evidence of such lesions.

**Significance:**

9.4‐Tesla MRI findings in epileptogenic lesions underlying DRFE are consistent with those on 3‐T MRI. UHF T2*‐weighted sequences may be useful to detect the black line sign and thereby refine surgical or ablation targeting for some FCDs. Assessment of the diagnostic yield of 9.4‐T MRI was limited by the lack of 3‐T MRI‐negative but histopathologically confirmed cases and by the unavailability of parallel transmit and FLAIR at 9.4 T. Further optimization of UHF protocols and analysis methods on larger cohorts may enhance clinically applicable diagnostic benefits.

**Key points**

- 9.4‐Tesla MRI shows FCD IIb with distinct qualitative and quantitative features that are consistent with 3‐T MRI.
- High‐resolution quantitative T2* maps at 9.4 T may provide additional information for defining resection or ablation targets in FCDs.
- Without using parallel transmit technology, artifacts in the temporal lobes pose a limitation of 9.4‐T MRI in presurgical epilepsy workup.
- FLAIR, key for detecting FCDs at 3 T, was not feasible at 9.4 T due to safety limits for radiofrequency‐induced tissue heating.

## 1 INTRODUCTION

In drug‐resistant focal epilepsy (DRFE), epilepsy surgery can achieve seizure freedom by resection or ablation of epileptogenic lesions. However, such lesions are often inconspicuous on magnetic resonance imaging (MRI) and may only be identified on histopathology.^1^, ^2^ Resection of MRI‐diagnosed lesions is associated with better clinical outcomes,^3^ whereas MRI‐negative patients need to undergo additional investigations such as intracranial electroencephalography (iEEG). Furthermore, as minimally invasive procedures, such as laser interstitial thermal therapy and radiofrequency ablation, are becoming more common,^4^ and limited resections of bottom‐of‐sulcus focal cortical dysplasias (FCDs) have been reported to be effective,^5^, ^6^ high‐resolution characterization of epileptogenic lesions is clinically necessary. Improving the sensitivity, specificity, and spatial precision of MRI to detect epileptogenic lesions is therefore expected to translate into direct clinical benefit in the management of DRFE patients.

FCDs represent one of the most common pathologies in DRFE. FCD type II lesions, the most common histopathological subtype, are characterized on MRI by cortical thickening, subcortical T2/fluid‐attenuated inversion recovery (FLAIR) hyperintensity extending to the ventricles (transmantle sign), and blurring of the interface of gray matter (GM) and white matter (WM).^7^ They can be subtle and hard to detect visually even for expert readers (with a detection rate of, e.g., 68%).^8^ The radiological features can be quantified by computational methods^9^, ^10^ and serve as inputs for (deep learning‐based) detection algorithms that may improve diagnostic yield in previously MRI‐negative cases.^11^, ^12^

Ultra‐high‐field (UHF) MRI is defined as magnetic resonance (MR)‐based imaging at magnetic field strengths of 7 T or above. Compared to 1.5‐ or 3‐T MRI, UHF MRI enables measurements with a higher signal‐to‐noise ratio (SNR),^13^ which can be traded for higher spatial resolution and/or shorter measurement duration. Furthermore, UHF MRI facilitates the acquisition of some contrasts like T2*‐weighted images. On the other hand, higher field strengths and the associated higher Larmor frequencies translate into reduced radiofrequency (RF) uniformity and increased RF power deposition, which results in artifacts and requires the adaptation of protocols.^14^

Consequently, UHF MRI can be expected to improve diagnostic sensitivity in DRFE. Small structural features may be captured with higher fidelity (e.g., blurring of the GM/WM border in FCDs or loss of hippocampal structure in hippocampal sclerosis). Several studies have employed 7‐T MRI for lesion detection in DRFE, reporting diagnostic gains (i.e., the ratio of patients with new relevant findings to previously MRI‐negative patients studied) of 20–30%.^15^, ^16^, ^17^, ^18^, ^19^, ^20^, ^21^ As a novel feature detectable by UHF MRI, the term “black line sign” has been coined to describe a focal cortical T2* hypointensity in FCD II.^22^, ^23^ Complete resection of the black line sign is associated with better surgical outcome. Although 7‐T MRI is becoming more widely used and is available as a CE‐certified medical product, only a few human 9.4‐T systems have been installed worldwide. 9.4‐Tesla MRI may offer yet another substantial gain in resolution and diagnostic power currently explored in basic science research. In vivo human measurements at even higher field strengths of, for example, 11.7 T are possible^24^ and may become more relevant in the future.

To the best of our knowledge, apart from preliminary data from this project,^25^ to date, only one case report of a focal epilepsy patient scanned at 9.4 T has been published.^26^ In addition, a protocol that includes 9.4‐T MRI on 7‐T MRI‐negative patients has been published, and the study is ongoing.^27^

Here, we present a cohort of DRFE patients and healthy controls examined using in vivo 9.4‐T MRI with standardized 3‐T MRI acquired for comparison. We investigated whether 9.4‐T MRI (1) increases diagnostic yield, that is, reveals epileptogenic lesions in 3‐T MRI‐negative patients; (2) reveals additional features or more intralesional detail, particularly in FCDs; and (3) enhances effect sizes of quantitative features characteristic of FCDs. These findings provide insights into the potential of UHF MRI to improve cortical profiling of FCDs and lesion detection in DRFE.

## 2 MATERIALS AND METHODS

### 2.1 Inclusion and exclusion criteria

Twenty‐one adult (>18 years old) patients with DRFE undergoing presurgical diagnostic workup and 20 healthy controls were recruited to participate in this observational diagnostic study. Exclusion criteria were the inability to provide informed consent, contraindications for UHF MRI (e.g., implants, claustrophobia, tattoos), and pregnancy or breastfeeding.

Recruitment and MRI examinations were conducted between March 2015 and October 2018. The study was approved by the local ethics committee (Medical Faculty of the University of Tübingen, reference number 390/2014BO1); participants and patients provided informed consent. In line with the ethics approval for this study, 9.4‐T MRI data were not used to inform clinical treatment decisions. The subject IDs used in this article (e.g., P012) are pseudonyms assigned exclusively for the purposes of this study; no information linking these identifiers to patients' identities or medical records was accessible to anyone outside the research group.

### 2.2 Demographic and clinical data

Demographic and clinical data were collected in the epilepsy surgery multidisciplinary team meetings at the University Hospital Tübingen. Postoperative outcomes and histopathology for surgically treated patients were gathered from the hospital electronic health record.

Patients had a median age of 27 years (range = 18–51); 11 were female, 10 male. Controls had a median age of 28 years (range = 21–50 years); seven were female, 13 male. For detailed information, see Table S1. Two of 21 patients (10%) had a clear finding on 3‐T MRI (*n* = 2 FCD, P017 and P026). Another three of 21 (14%) had a suspected epileptogenic lesion (*n* = 2 FCD, P014, P016; *n* = 1 hippocampal sclerosis, P009) reported in at least one but not each of the clinical MRI examinations. The remaining patients (76%) were 3‐T MRI‐negative. With respect to noninvasive video‐EEG, which was conducted in all patients, seizures were recorded in all but one patient. Thirteen of 21 patients (62%) underwent 18F‐fluorodeoxyglucose positron emission tomography, and two of 13 had localizing concordant findings. Intracranial EEG was performed in six of 21 (29%). Three of 21 patients (14%, P014, P017, P026) underwent surgical resections; histopathology revealed FCD IIb in two of three (P017, P026) and only fragmented tissue without evidence of pathology in one of three (P014). The operated patients with FCD IIb (P017, P026) had good clinical outcomes (Engel class I).

### 2.3 MRI data acquisition

9.4‐Tesla MRI scans were acquired with a human whole‐body 9.4‐T MRI scanner (Siemens Healthineers) located at the Department for High‐Field Magnetic Resonance of the Max Planck Institute for Biological Cybernetics, Tübingen. A custom built head coil with a 16‐element dual row transmit array and a 31‐element receive array was used.^28^ MRI sequences included the following: (1) whole brain .8‐mm isotropic magnetization‐prepared two rapid acquisition gradient echoes^29^ (MP2RAGE; echo time [TE] = 2.3 ms, repetition time [TR] = 6 ms, inversion time 1 [TI1] = 900 ms, TI2 = 3500 ms, flip angle 1 [FA1] = 4°, FA2 = 6°, volume TR = 9 s); and (2) a T2*‐weighted monopolar multiecho gradient echo (GRE) with .375 × .375 × .8 mm^3^ voxel size (TE = 6–30 ms in steps of 6 ms, TR = 35 ms, FA = 11°) acquired in slabs positioned according to the suspected lesions or clinical hypotheses. 9.4‐Tesla MP2RAGE UNI and T1 maps were reconstructed offline with correction for excitation FA and inversion efficiency deviations.^30^ Derived, synthetic inversion‐recovery contrasts expected at TI = 900, 1200, and 1500 ms were generated from the quantitative MP2RAGE T1 maps from the following relationship: abs(1–2 exp.[T_I_/T_1_]). Derived fluid and WM suppressed (FLAWS) MR images were generated after optimization of the TIs at 9.4 T.^31^ Multiecho GRE data were processed as described previously^32^, ^33^ to generate T2* and quantitative susceptibility mapping (QSM) maps.

3 T MRI scans were collected with a MAGNETOM Prisma scanner (Siemens Healthineers) with a 64‐channel head coil. The following data were acquired: each whole brain 1.0‐mm isotropic (1) T1‐weighted magnetization‐prepared rapid acquisition gradient echo (MPRAGE; TE = 2.98 ms, TR = 2300 ms, TI = 900 ms, FA = 9°), (2) MP2RAGE (TE = 2.98 ms, volume TR = 5000 ms, TI1 = 700 ms, TI2 = 2500 ms, FA1 = 4°, FA2 = 5°), and (3) T2‐FLAIR weighted sequence (sampling perfection with application optimized contrasts using different FA evolution–FLAIR; TE = 388 ms, TR = 5000 ms, TI = 1800 ms, FA = 120°).

### 2.4 Visual evaluation

9.4‐Tesla and 3‐T MR images were reviewed by a neuroradiologist (B.B.) and two neurologists (C.K., P.M.) with expertise in imaging of focal epilepsies for possible epileptogenic lesions or other incidental findings. Reviewers were not blinded and had access to clinical information. Qualitative findings were recorded.

### 2.5 Lesion masks

To guide the extraction of features by surface‐based morphometry, lesions were manually labeled. For operated patients, preoperative (9.4 T and 3 T) as well as clinical postoperative images were used to define lesion masks using FreeView software (C.K. and P.M.).

### 2.6 Surface‐based analysis

Details are described in Supplementary Methods. Briefly, MP2RAGE images were denoised^34^ and bias‐field corrected prior to segmentation and cortical surface reconstruction using FreeSurfer^35^ 7.4.1 at native (i.e., for 9.4 T: submillimetric) resolution with synthstrip^36^ skull stripping. Available volumetric data (at 3 T: MP2RAGE, T1 maps, normalized MPRAGE, normalized FLAIR; at 9.4 T: MP2RAGE, T1 maps, T2* maps, QSM maps) were projected onto the cortical surface mesh at six depths ranging from −1.0 to −6.0 mm below the pial surface. Surface meshes and lesion masks were registered to a bilaterally symmetric template (fsaverage_sym) to enable per‐vertex comparisons. Homotopic contralateral regions served as within‐patient controls. The median of these quantitative features across the entire lesion mask or in bins defined according to the distance from the lesion centroid was calculated and *z*‐scored relative to the healthy control distribution.

#### 2.6.1 High‐resolution profiling of T2* data

The FreeSurfer cortical segmentation within the manually labeled lesion and in the surrounding cortex up to a geodesic distance of 100 mm from the lesion centroid was processed using LayNii^37^ to assign each voxel to a cortical layer (equivolumetric layering, with an arbitrarily chosen number of 10 bins) and to a cortical column (average column volume of 40 mm^3^). Columnwise profiles of T2* in different cortical depths were extracted. Based on exploratory analyses and visual verification that columns were selected from anatomically appropriate locations, a profile was classified as consistent with a black line sign if it had a T2* value < 30 ms in layer 5 and T2* > 30 ms in layer 2. The average profile of columns meeting these criteria was extracted and used as a target profile. For each column, the root mean square error between its T2* profile and the target profile was calculated as a measure of similarity, log‐transformed for visualization, and overlaid onto the T2* maps as well as plotted against geodesic distance from the lesion centroid in a scatterplot. This analysis was repeated after downsampling T2* maps to 1.0‐mm isotropic spatial resolution (using NiBabel 5.3.2, with linear interpolation).

#### 2.6.2 Comparison of surface‐based image features across field strengths

To compare image features between field strengths across patients and controls, we selected mean cortical thickness as a morphological feature and MP2RAGE apparent contrast to noise ratio (aCNR) in GM and WM as an intensity feature. We aggregated these features by averaging across the whole cortex subjectwise and computed a mixed‐effects linear regression model with field strength (9.4 T/3 T), group (patient/control), and their interaction as categorical fixed effects and subject as random effect. We also assessed the spatial differences according to the Desikan–Killiany cortical parcellation. For details, see Supplementary Methods.

### 2.7 Data and code availability

The healthy volunteer 9.4‐T MP2RAGE data have been published as part of a larger dataset.^38^ It is currently not possible to make the patient imaging data openly accessible due to data protection regulations. The code used for analysis is available at https://github.com/ckronlage/3T9T_profiling_pub.

## 3 RESULTS

### 3.1 Visual evaluation of MR images

In the *n* = 16 MRI‐negative patients, no new epileptogenic lesions concordant with clinical hypotheses were identified upon visual inspection.

Two histologically verified FCD lesions that were visible at 3 T (Patients P017 and P026) were clearly discernible at 9.4 T (see Figures 1 and 2), characterized by increased cortical thickness on T1‐weighted images. In one of the lesions, blurring of the GM/WM border was visible, which was more conspicuous in 9.4‐T MP2RAGE. Subcortical T2/FLAIR hyperintensity (transmantle sign) in the 3‐T data was visible in Patient P017 and less visible in Patient P026. A similar hyperintensity was visible in the 9.4‐T GRE_TE1_ magnitude image but less visible in the T2* map in Patient P017 (see Figure 1). Additionally, a subtle intracortical region of decreased T2* values was identified. Synthetic contrasts derived from the 9.4‐T T1 map showed subtle abnormalities of the GM/WM border and the subcortical WM, although with limited specificity (Figure S1).

**FIGURE 1:**
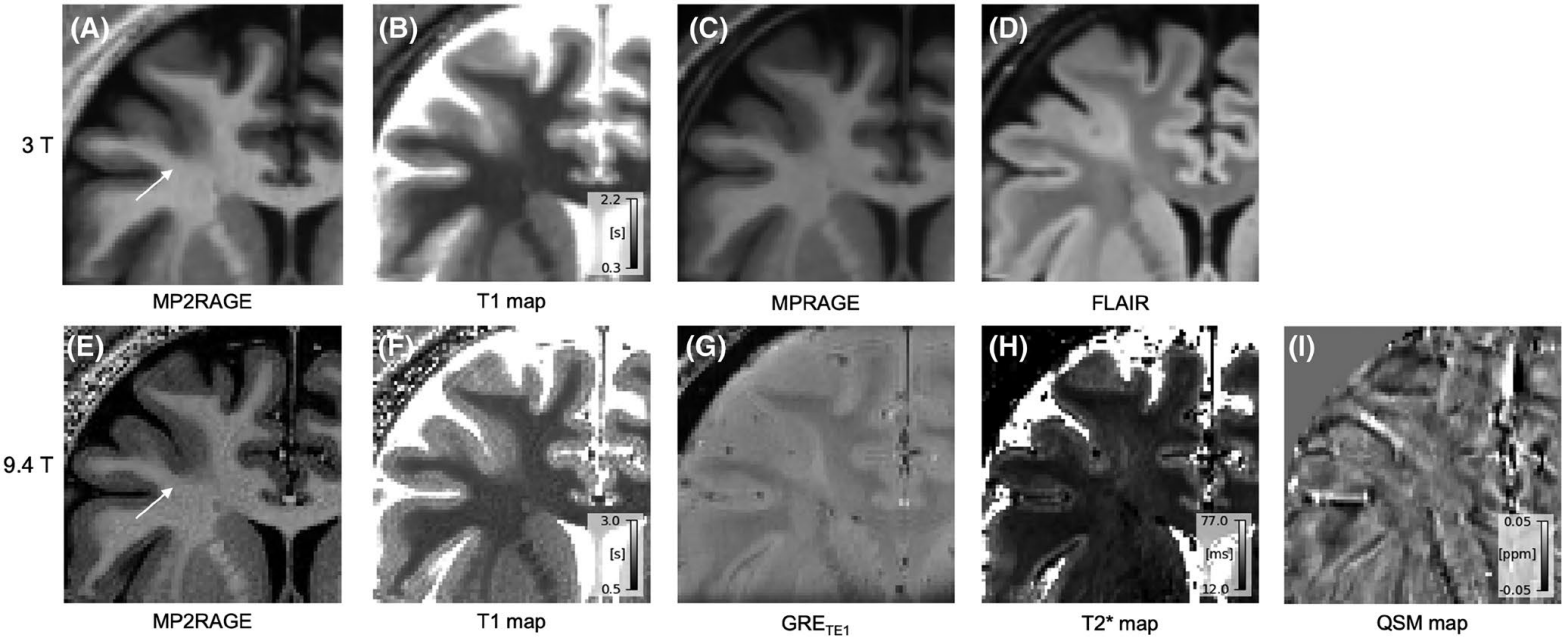
Focal cortical dysplasia (FCD) IIb lesion in Patient P017. Coronal slices are shown, with comparison of 3‐T data (top row, A–D) and 9.4‐T data (bottom row, E–I). Thickening of the cortex, blurring of the gray matter (GM)/white matter (WM) interface, and subcortical T2/fluid‐attenuated inversion recovery (FLAIR) hyperintensity (transmantle sign) are visible on the 3‐T images as typical imaging features of an FCD. Increased cortical thickness and reduced sharpness of the GM/WM border are discernible on the 9.4‐T images as well. The echo time 1 gradient echo (GRE_TE1_) shows subcortical hyperintensity; correspondingly, there are higher T2* values. A stripe of decreased T2* in the cortex in the center of the lesion (possibly corresponding to the “black line sign” described previously^22^, ^23^) is seen. The quantitative susceptibility mapping (QSM) shows heterogeneous signal without clear contrast between the lesion and neighboring areas or GM and WM. MP2RAGE, magnetization‐prepared two rapid acquisition gradient echoes; MPRAGE, magnetization‐prepared rapid acquisition gradient echo.

**FIGURE 2:**
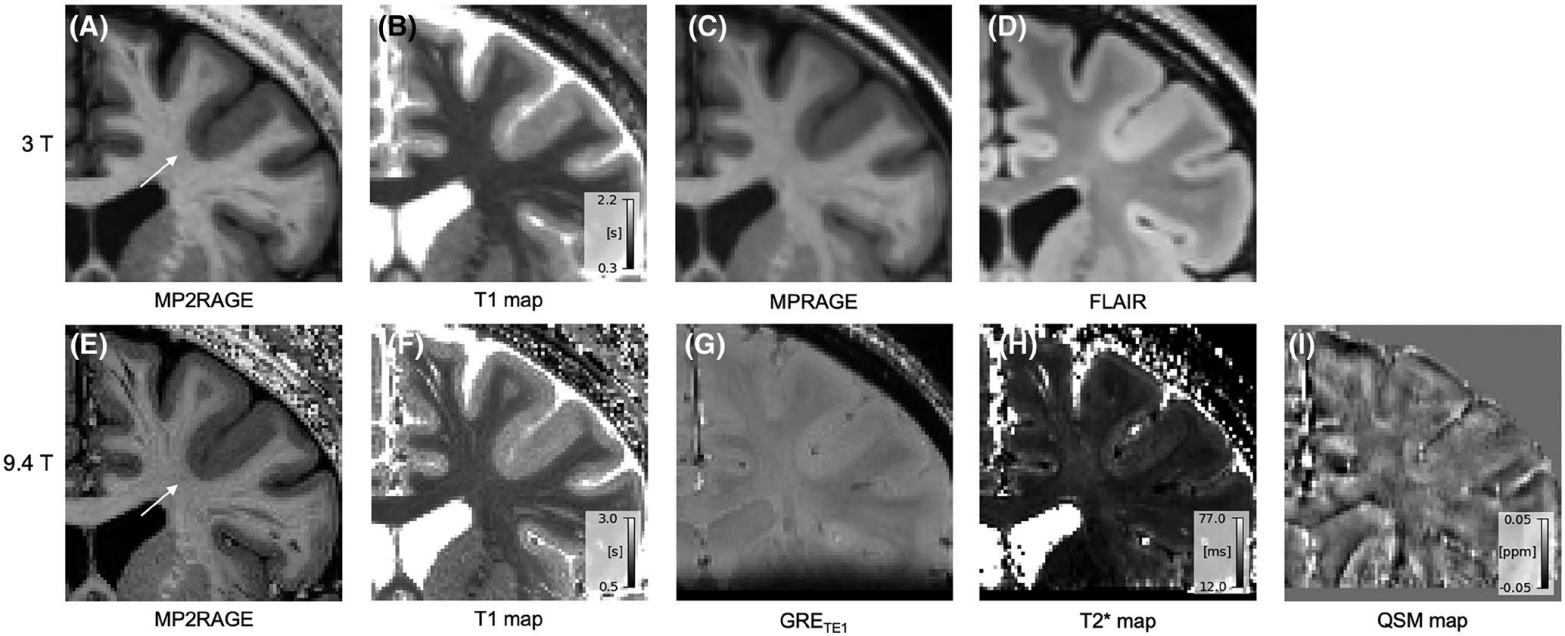
Focal cortical dysplasia IIb lesion in Patient P026. Coronal slices are shown, with comparison of 3‐T data (top row, A–D) and 9.4‐T data (bottom row, E–I). On the 3‐T images, thickening of the cortex is visible; in the fluid‐attenuated inversion recovery (FLAIR) image, a subtle subcortical FLAIR hyperintensity can be suspected. There is no blurring of the gray matter/white matter border. In the 9.4‐T data, the same imaging features (cortical thickening without blurring) are apparent. The echo time 1 gradient echo (GRE_TE1_) and T2* data do not show a subcortical signal comparable to the 3‐T FLAIR. There is no clear alteration of quantitative susceptibility mapping (QSM) in the lesion. MP2RAGE, magnetization‐prepared two rapid acquisition gradient echoes; MPRAGE, magnetization‐prepared rapid acquisition gradient echo.

In the patient with suspected right hippocampal sclerosis (see Figure S2), hippocampal volume loss was visible on both 3‐T and 9.4‐T MRI T1‐weighted images. There was no clear T2/FLAIR hyperintensity visible in 3‐T MR images. The findings were not discernible on 9.4‐T T2*‐weighted GRE images or derived T2* maps. In patients with suspected but unconfirmed FCDs (P014, P016), no qualitatively new findings were identified at 9.4 T (see Figure S3).

### 3.2 9.4‐Tesla MR image quality

All 9.4‐T MP2RAGE volumes contained minor artifacts in the lower temporal lobes. Three T1‐weighted volumes (from two patients and one control) contained more pronounced artifacts impeding delineation of entire temporal cortical gyri and sulci (see examples in Figure S4). However, both FCDs profiled in more detail were outside these low‐SNR areas.

### 3.3 Surface‐based profiling of histologically verified FCDs

Two patients, P017 and P026, had histologically verified FCDs. Surface reconstructions in the lesion areas appeared visually accurate for both field strengths and showed high agreement (see Figure 3A–C,E–G). Estimates of cortical thickness in the lesion region of interest (ROI) were comparable between 3‐T and 9.4‐T MR images (numerically slightly larger for 9.4‐T compared to 3‐T data for P017, and slightly reduced for P026, median 3.84 vs. 3.94 mm and 3.07 vs. 3.02 mm, respectively; see Figure 3D,H). For both 3‐T and 9.4‐T MR images, cortical thickness values were clearly above average compared to controls when sampled at homotopic regions (see Figure 3D,H).

**FIGURE 3:**
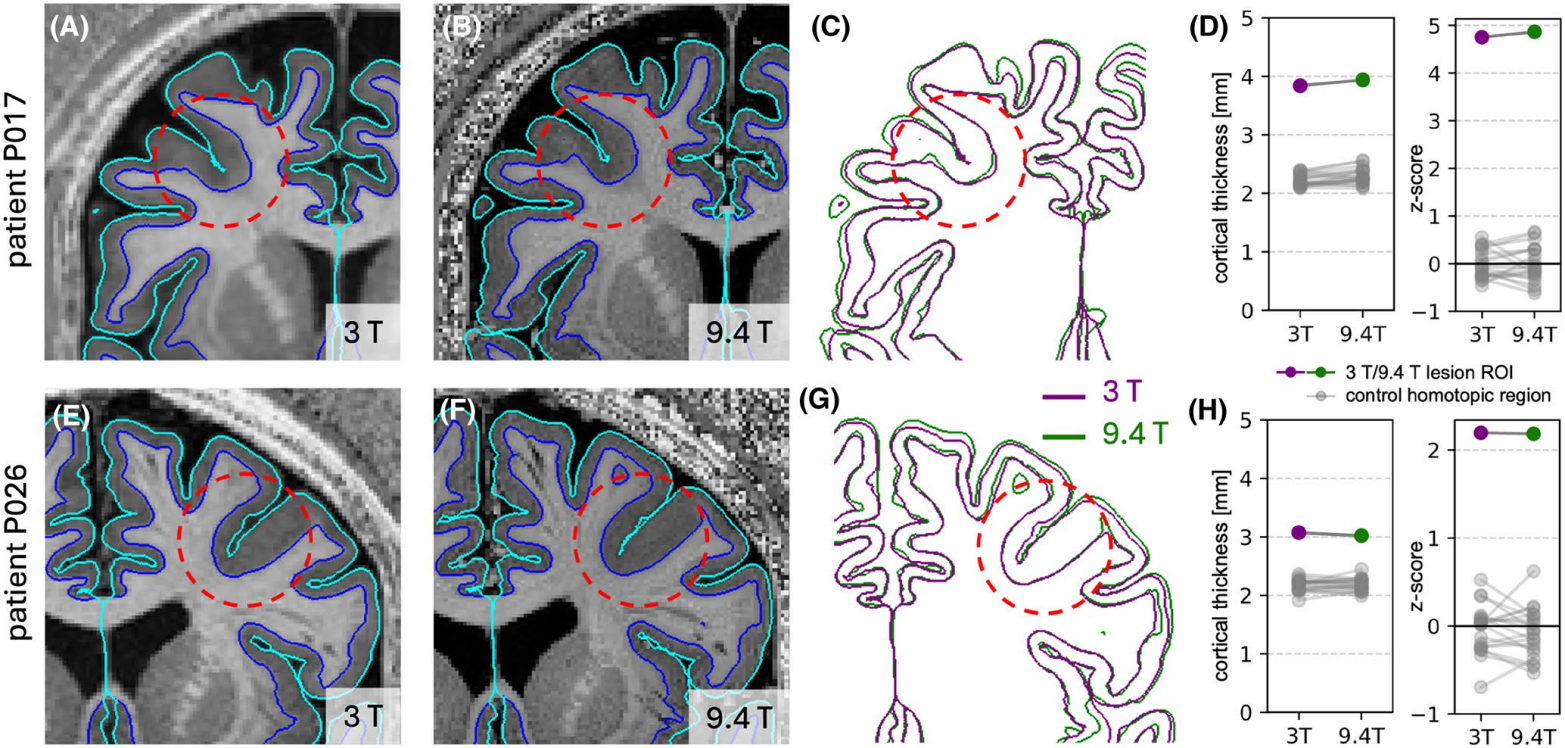
Cortical surface reconstructions overlaid on coronal slices of magnetization‐prepared two rapid acquisition gradient echoes uniform volumes (pial surface: cyan, gray matter/white matter boundary: blue). Top row (A–D): Patient P017. Bottom row (E–H) Patient P026. The respective lesion areas are highlighted with red circles. (A, E) Three‐tesla images. (B, F) 9.4‐Tesla images. (C, G) Overlay of coregistered surface outlines. (D, H) Plot of median lesion cortical thickness; raw values are shown on the left, *z*‐score normalized in reference to healthy controls (plotted in gray) on the right. Visually, there are few differences between reconstructions in the lesion areas. ROI, region of interest.

Both lesions show a distinct depth‐dependent profile with regard to some but not all extracted features (see Figure 4). Specifically, increased normalized FLAIR intensity, increased quantitative T1 (qT1) values, and increased T2* values at 3–6 mm of subpial depth were distinctive features. In contrast, there was no clear effect for estimates based on QSM within and below the lesion area. A direct numerical comparison between 3‐T and 9.4‐T MP2RAGE‐based qT1 values revealed a maximum *z*‐score of 2.8 at 3 T and 2.9 at 9.4 T for P017, and 1.3 at 3 T and 1.7 at 9.4 T for P026. As overall cortical thickness was lower at 9.4 T (see below), we also computed the ratio of mean lesion thickness to mean control cortical thickness as 172% (3 T) versus 188% (9.4 T) for P017, and 137% (3 T) versus 144% (9.4 T) for P026. The ratio of lesion thickness to mean thickness in all nonlesional cortex of the same patient was 186% (3‐T MRI) versus 201% (9.4‐T MRI) for P017 and 141% (3‐T MRI) versus 145% (9.4‐T MRI) for P026. Statistical tests could not be calculated, as only two patients were analyzed.

**FIGURE 4:**
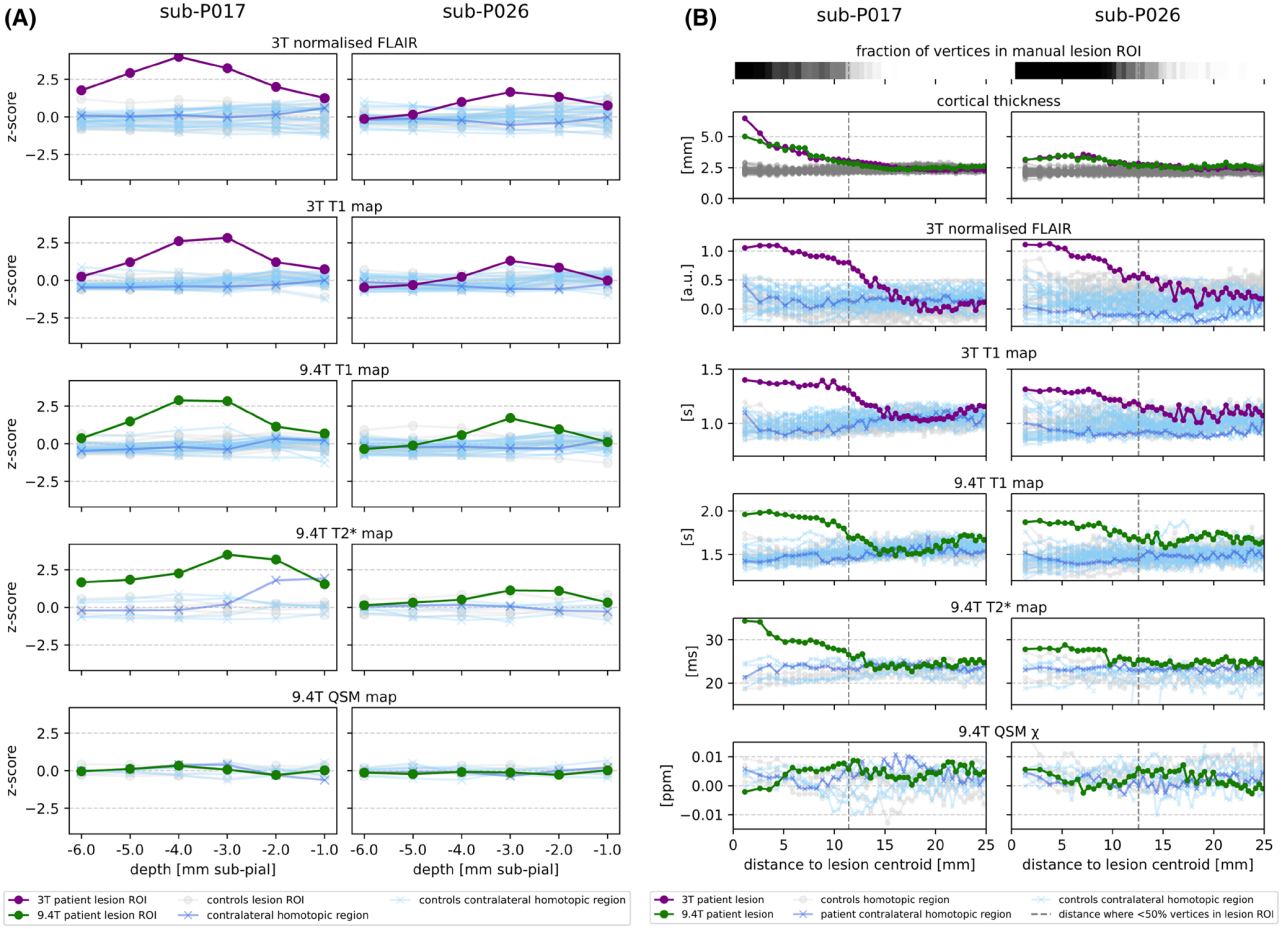
Vertical and horizontal quantitative profiles of two histologically verified focal cortical dysplasias across magnetic field strengths of 3 T and 9.4 T. For comparison, features in the contralateral homotopic region of patients are shown with crosses in blue, data from controls in light gray and blue. (A) For each feature, the median *z*‐scored value (relative to controls) in the lesion region of interest (ROI) is plotted against subpial depth. It is visible that both lesions show fluid‐attenuated inversion recovery (FLAIR) hyperintensity, higher T1 values, higher T2* values, and no clear changes in susceptibility. The largest *z*‐scores were obtained for 3‐T FLAIR. 3 T and 9.4‐T quantitative T1 have similar effect sizes. Raw, non‐*z*‐scored data are shown in Figure S5. (B) Horizontal profiles of different features, in bins with equal number of vertices in increasing distance from the centroid of the manually defined lesion label. The intensity features (FLAIR, T1, T2*, quantitative susceptibility mapping [QSM]) were sampled at 3‐mm subpial depth. The topmost bar shows the fraction of vertices inside the manually defined lesion label in grayscale; the distance where the fraction is .5 is marked with a vertical dashed line. The lesion extent appears similar for data acquired at both magnetic field strengths. sub, subject.

When inspecting the data with a focus on horizontal lesion extent (i.e., parallel to the cortical surface), for both lesions, differences compared to controls were apparent for all features except for QSM, up to approximately 10–15 mm from the ROI centroid (see Figure 4B). This aligns with the extent of the manually drawn lesion labels.

We quantitatively examined the reduced midcortical T2* values in the center of the FCD in Patient P017 (see Figure 5), corresponding to the previously described black line sign,^22^, ^23^ by defining a high‐resolution cortical profile in the T2* maps with .375‐mm in‐plane resolution. A midcortical decrease in T2* values was apparent in quantitative profiles of columns at the bottom of the sulcus (see Figure 5C–E). We observed a distance‐dependent decrease of profile similarity to the average target profile from the center of the lesion to further outward (see Figure 5F‐H). When applying the same target profile to the other FCD lesion (Patient P026) without a visually apparent black line sign, we did not observe the same pattern (see Figure S6). We repeated the layer‐based analysis of the black line region in Patient P017 after resampling the data to a lower 1.0‐mm isotropic resolution (Figure 5I‐P). Here, only two (rather than three) cortical columns met the criteria that we defined for the sign. The derived target T2* profile appeared to be less specific in the resampled data (Figure 5N–P).

**FIGURE 5:**
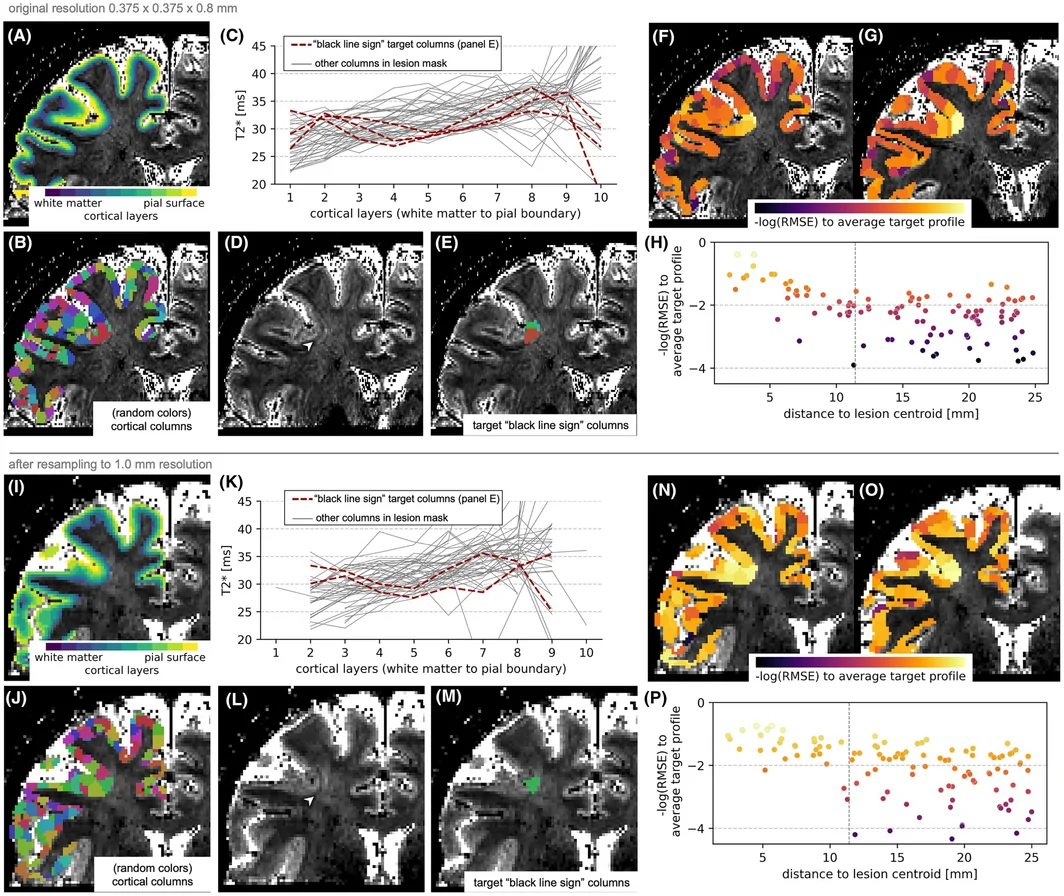
High‐resolution layer‐wise analysis of T2* at 9.4 T in a histologically verified focal cortical dysplasia IIb. Cortical voxels were assigned to 10 equivolumetric cortical layers (A) and cortical columns with a volume of 40 mm^3^ each (B) using the LayNii software suite. Profiles of T2* versus cortical layer were extracted. (C) Profiles of all columns in the manual lesion mask. Columns overlapping with the visually apparent black line sign (white arrowhead in D, raw T2* map) were selected according to manually set criteria (see Materials and Methods) and are shown in panel E. These selected target columns' profiles are colored in red in panel C, whereas other columns' profiles (within the lesion mask, but outside the black line sign area) are plotted in gray. In a next step, for each column, as a measure of similarity of its T2*profile to the average target profile, the log‐transformed root mean square error (RMSE) was computed. These values are plotted for two coronal slices (F, G) and plotted against the distance from the lesion centroid, with the dashed line representing the border of the manual lesion region of interest as in Figure 4B (H). Columns within 5‐mm distance from the lesion centroid are more similar to the target black line sign T2* profile, whereas columns at >5‐mm distance are less similar and exhibit higher variability. (I–P) Results of the same analysis after downsampling the data to 1.0‐mm isotropic resolution. The black line sign can still be identified by quantitative profiling, although only two columns rather than three (with the same volume of 40 mm^3^ each) fulfill the set criteria, and specificity of this profile in comparison to surrounding areas is lower (N–P).

### 3.4 Comparison of cortical image features across 9.4 T and 3 T

Across all subjects, cortical thickness estimates were thinner at 9.4 T than 3 T (median difference = .14 mm, mixed‐effects linear regression beta = −.15, *p* < .001). The strongest differences in cortical thickness measurements appeared in the temporal lobes. Apparent aCNR between GM and WM in MP2RAGE UNI images, without correction for different voxel volumes, was lower at 9.4 T than 3 T (median difference = .20, beta = −.22, *p* < .001). Spatially, significant differences were mainly present in the lower temporal lobes, as well as in the central and superior temporal regions (Figure S7).

## 4 DISCUSSION

We present data from *n* = 21 focal epilepsy patients and *n* = 20 healthy controls scanned at 9.4‐T MRI and compare them to 3‐T MRI data in the same participants. Overall, epileptogenic lesions were visible at both field strengths. Two of the recruited patients with previous 3‐T MRI findings had FCDs that were subsequently histopathologically verified. For FCDs, 9.4‐T MRI was able to recover features classically seen at 3 T, some with increased detail due to higher resolution. Specifically, in one FCD, there was a visible and quantifiable intralesional microstructural heterogeneity (reduced intracortical T2* values, reflective of the black line sign) that we quantified using an automated approach. Lesions missed on 3‐T MRI and subsequently confirmed histologically were not present in this cohort, although such cases would be particularly informative for UHF MRI analysis. This study provides new details on how UHF MRI data can be used to quantitatively profile cortical pathology.

Surface‐based morphometry features quantitatively characterize FCDs^10^ and are used by research tools that generate automatic predictions for FCD localization.^11^, ^12^ However, to date, none of these approaches has been optimized or evaluated for UHF MRI data. In this study, we apply a surface‐based reconstruction with preprocessing steps adapted for the MP2RAGE 9.4‐T data and compare quantitative features of FCDs across 9.4‐T and 3‐T MR images. Depth‐dependent changes and horizontal lesion spread can be measured at both field strengths (see Figure 5).

UHF MRI enables optimized T2*‐weighted imaging and quantitative mapping of T2* as well as susceptibility (QSM).^39^ In FCD IIb, a focal hypointensity on 7T T2*‐weighted images in the center of the lesion has been termed black line sign.^22^, ^23^ It has been found to be concordant with the seizure onset zone identified by iEEG. Furthermore, resection of the respective region was associated with better surgical outcomes. Here, we observe a similar alteration on 9.4‐T T2* maps in one of two type IIb FCDs. We also indicate that this pattern can be resolved quantitatively with high‐resolution cortical profiling, which motivates future research to extract this feature automatically from UHF MRI data. UHF MRI may thus have a role in increasing the precision of the MRI‐defined epileptogenic lesion and neurosurgical resection or ablation target.

As we did not acquire a comparable T2*‐weighted sequence at 3 T, we cannot directly evaluate whether the black line sign would also have been visible at lower field strengths. To our knowledge, no direct comparisons between UHF and 3T T2*‐weighted images have been reported, but only the retrospective identification with reduced conspicuity of a part of these findings on 3‐T T2‐weighted and FLAIR images.^23^ In our case, a 3‐T FLAIR sequence did not show corresponding signal changes (Figure 1D). As shown by downsampling the data (Figure 5I–P), high spatial resolution of 9.4‐T T2* images is a contributing factor in detecting the sign, but intrinsically higher T2* contrast at higher field strengths may also play a role.^14^ Similarly, for detecting the central vein sign in multiple sclerosis, T2*‐weighted 7‐T MRI has demonstrated higher sensitivity than 3‐T MRI.^40^

A previous 3‐T study of 18 histopathologically confirmed FCDs found decreased QSM χ values in FCD IIb, most pronounced at 2–4‐mm subpial depth, but no R2* changes.^41^ We observed a different pattern for the two type IIb FCDs: increased T2* and no QSM change at 9.4 T, suggesting altered T2* rather than susceptibility. Interpretation is complicated by a recent observation of widespread alterations in qT2 in focal epilepsy even distant from the seizure onset.^42^ An ex vivo study using quantitative histology and 9.4‐T MRI attributed changes in T2 and T2* in a type IIa FCD and changes in T1, T2, and T2* in a type IIb FCD mainly to reduced myelination and altered neuronal density.^43^

In contrast to previous work that compared 7‐T with 3‐T MRI, we could not identify new, previously undetected epileptogenic lesions using 9.4‐T MRI in this cohort. Given there were also no histologically verified lesions in 3‐T MRI‐negative patients, it is unknown whether more lesions existed. The diagnostic gain (i.e., fraction of MRI‐negative cases with new relevant findings) of 7‐T MRI has been reported as 84 of 205 (41%) in a systematic review of studies up to 2021,^16^ and in more recent studies as three of 8 (38%),^17^ four of 24 (17%),^18^ four of 21 (19%),^19^ nine of 31 (29%),^15^ nine of 60 (15%),^20^ and six of 26 (23%).^21^ In a single reported case of 9.4‐T MRI in a 3‐T and 7‐T MRI‐negative focal epilepsy patient, no new lesion was identified.^26^ This discrepancy is likely due to methodological limitations. With 21 patients, although comparable to some of the cited studies, our cohort is relatively small.

In addition, at the time of data acquisition, it was not yet feasible to acquire a standard FLAIR sequence at 9.4 T due to specific absorption rate limitations. However, FLAIR is one of the most relevant contrasts for detecting hippocampal sclerosis and FCDs.^10^, ^44^ Using 7‐T MRI, FLAIR sequences can already be acquired, and FLAIR features, like the transmantle sign in FCD IIb, are important for identifying new lesions.^21^ Moreover, RF nonuniformity and susceptibility effects are more pronounced at 9.4 T and can cause visible artifacts, especially in the lower temporal lobes (see Figure S4). This, too, could have prevented the detection of new lesions in this area. Parallel transmit technology can mitigate some of these issues but was not yet robustly available at 9.4 T during this study. Further methodological innovations may improve 9.4‐T UHF image acquisitions in the future (see more extended discussion on this point in Supplementary Material).

SNR scales supralinearly with field strength^13^ and linearly with voxel volume, and with the square root of acquisition time.^45^ CNR, however, also depends on tissue properties and different contrast mechanisms.^46^ In our data, we found a slightly lower cortical GM‐to‐WM aCNR in 9.4‐T compared to 3‐T MRI data (see Figure S7) without correction for the smaller voxel volume at 9.4 T. This finding is consistent with previous comparisons that reported lower uncorrected SNR and similar CNR for many sequences at 7 T versus 3 T, although overall diagnostic confidence was still rated higher based on 7‐T MRI data.^47^ Lower cortical thickness estimates at UHF are also in line with findings at 7 T.^48^ For both features, differences were most pronounced in the temporal lobes, so they may be caused by RF inhomogeneity effects (Figure S7). These quantitative comparisons between 3‐T and 9.4‐T MRI data highlight differences in cortical features extracted from the same participants and patients, implying that both visual inspection criteria and automated algorithms have to be adapted for UHF in the future.

In conclusion, imaging characteristics of FCDs at 3 T can also be detected at 9.4 T. Quantitative feature profiles in T1‐ and T2*‐weighted sequences differentiate lesional cortical and subcortical areas from homotopic areas in patients and healthy controls. High‐resolution T2*‐weighted imaging can provide additional diagnostic information, such as by identifying the black line sign that has previously been shown to be concordant with the seizure onset zone. This exploratory study does not demonstrate an increased diagnostic sensitivity using 9.4‐T MRI with respect to the detection of lesions in 3‐T MRI‐negative patients, although it is limited by the inclusion of only two patients with histopathologically verified lesions and by the usage of 9.4‐T MRI data with minor artifacts in the temporal lobes. Future work will include applying and adapting computational tools for automated lesion detection to the 9.4‐T MRI data.

## AUTHOR CONTRIBUTIONS

**Cornelius Kronlage:** Conceptualization; investigation; methodology; formal analysis; writing—original draft preparation; writing—review and editing. **Pascal Martin:** Conceptualization; methodology; investigation; writing—review and editing. **Benjamin Bender:** Investigation; writing—review and editing. **Gisela E. Hagberg:** Investigation; methodology; formal analysis; writing—review and editing. **Jonas Bause:** Investigation; methodology; writing—review and editing. **Joana R. A. Loureiro:** Investigation; methodology; writing—review and editing. **Mathilde Ripart:** Methodology; writing—review and editing. **Sophie Adler:** Methodology; writing—review and editing. **Konrad Wagstyl:** Methodology; writing—review and editing. **Holger Lerche:** Resources; writing—review and editing. **Niels K. N. Focke:** Conceptualization; methodology; writing—review and editing. **Esther Kuehn:** Conceptualization; investigation; writing—review and editing.

## FUNDING INFORMATION

We acknowledge the following funding: MINT‐CS (Faculty of Medicine Tübingen, Deutsche Forschungsgemeinschaft grant 01GQ2101; C.K.), PRECISE.net (Else Kröner Fresenius Stiftung; C.K.), de.NBI Cloud within the German Network for Bioinformatics Infrastructure (de.NBI) and ELIXIR‐DE (Forschungszentrum Jülich and W‐de.NBI‐001, W‐de.NBI‐004, W‐de.NBI‐008, W‐de.NBI‐010, W‐de.NBI‐013, W‐de.NBI‐014, W‐de.NBI‐016, W‐de.NBI‐022; C.K), the Wellcome Trust (grant 301991/Z/23/Z; M.R., K.W.), the Epilepsy Research Institute (P2208; S.A.), EU‐LACH (grant #16/T01‐0118; G.E.H.), and BMBF (grant 01GQ2101; G.E.H.).

## CONFLICT OF INTEREST STATEMENT

None of the authors has any conflict of interest to disclose. We confirm that we have read the Journal's position on issues involved in ethical publication and affirm that this report is consistent with those guidelines.

## Supporting information


**FIGURE S1.** Comparison of acquired and derived edge‐enhancing gradient echo (EDGE) inversion recovery contrasts and fluid and white matter suppression (FLAWS) in focal cortical dysplasias. Top row (A–F): Patient P017; bottom row (G–L): Patient P026. (A, G) Three‐tesla magnetization‐prepared 2 rapid acquisition gradient echo (MP2RAGE) inversion time 1 image (INV1). (B, H) 9.4‐Tesla MP2RAGE INV1. (C–E, I–K) Derived synthetic inversion recovery (IR) contrasts generated based on 9.4‐T MP2RAGE T1 map with theoretical inversion times (TIs) of 900, 1200, and 1500 ms, respectively. (F, L) Derived FLAWS contrast. In the 3‐T INV1, there is similar signal intensity in white and gray matter and a hypointense gray–white border, similar to what has previously been termed EDGE.^1,2^ At 9.4 T, sequence parameters had not been optimized for this contrast, so that the acquired 9.4‐T INV1 shows hyperintense gray matter. However, it is possible to generate a derived IR contrast in postprocessing with different TIs, where a TI of approximately 1200 ms results in an EDGE‐like contrast, with smaller voxel sizes at 9.4 T and better delineation of the cortex. Interestingly, EDGE‐like contrasts show slight blurring and widening of the gray–white matter boundary in Patient P017 (A, D). Derived FLAWS^3^ images show subtle subcortical hyperintensity in Patient P017 (F).
**FIGURE S2.** Coronal slices Patient P009 with suspected right hippocampal sclerosis. Comparison of 3‐T data (top row, coregistered and resampled to the 9.4‐T volume with cubic interpolation) and 9.4‐T data (bottom row). Volume loss of the right hippocampus and corresponding enlargement of the temporal horn of the lateral ventricle can be appreciated in the 3‐T images. There is no clear T2/fluid‐attenuated inversion recovery (FLAIR) hyperintensity. Right hippocampal volume loss is also clearly visible in the 9.4‐T image, with more sharply defined borders. 9.4‐T gradient echo (GRE) and derived maps (T2*, quantitative susceptibility mapping [QSM]) do appear to show a contrast in this area and are affected by artifacts in proximity to the skull base.
**FIGURE S3.** Axial slices of the two suspected focal cortical dysplasia (FCD) in Patient P016 where no seizures could be recorded on video‐electroencephalography and a differential diagnosis of nonepileptic seizures was discussed (also rendering the diagnosis of FCDs uncertain). Top rows: An asymmetrical T2/fluid‐attenuated inversion recovery (FLAIR) hyperintensity is visible in the posterior right insular cortex, in the 9.4‐T gradient echo (GRE) a similar finding can be suspected, and the T1‐weighted sequences reveal no clear abnormality. Bottom rows: A blurring of the gray matter–white matter boundary and subcortical FLAIR hyperintensity is visible in the occipital sulcus in the 3‐T data.
**FIGURE S4.** Artifacts in the temporal lobes in the 9.4‐T magnetization‐prepared rapid acquisition gradient echo (MPRAGE) data. Left: Example of a more pronounced artifact (as present in 3/41 subjects in total). Right: Example of a less pronounced artifact as present in all of the subjects. Top row: MP2RAGE uniform volumes; middle row: MP2RAGE INV1; bottom row: MP2RAGE INV2. These findings are likely mainly caused by transmit field inhomogeneities.
**FIGURE S5.** Vertical profiles of two histologically verified focal cortical dysplasias across magnetic field strengths of 3T and 9.4T. For each feature and depth, the raw feature value in the lesion region of interest (ROI) is plotted. Also see Figure 4.
**FIGURE S6.** High‐resolution layerwise analysis of T2* at 9.4 T in a histologically verified focal cortical dysplasia IIb in Patient P026. Cortical voxels were assigned to 10 equivolumetric cortical layers (A) and cortical columns with a volume of 40 mm^3^ each (B) using the LayNii software suite. Profiles of T2* versus cortical layer were extracted. (C) Profiles of all columns in the manual lesion mask. In this case, there were no columns matching the manually set criteria (see Materials and Methods) for the “black line sign” feature. (D) T2* map without overlays. We assessed the similarity of Patient P026 column T2* profiles to the target profile derived from Patient P017. The log‐transformed root mean square error (RMSE) is plotted overlaid onto the respective columns (E) and against the distance from the lesion centroid (F). In this case, although individual columns have a higher similarity score, there is no consistent distance‐dependent pattern as in Patient P017.
**FIGURE S7.** Comparison of cortical thickness (A–D) and white/gray matter apparent contrast to noise ratio (aCNR; E–H) between paired 9.4‐T and 3‐T magnetization‐prepared rapid acquisition gradient echo acquisitions. The plots show these data aggregated in different ways. (A, E) Bland–Altmann plots of paired 9.4‐T and 3‐T measurements averaged per subject: difference on the y‐axis, mean on the x‐axis, kernel density estimate plot of the difference marginal distribution on the right, colored according to group (patient vs. control). For both thickness and aCNR, a mixed‐effects linear regression model identified a significant effect of field strength (9.4 T/3 T, *p* < .001), whereas group label (patient/control) and its interaction with field strength interaction were not significant. (B, F) Median difference per parcellation region of interest (ROI) for each feature overlaid onto a template inflated surface plot, only shown if Wilcoxon test *p* < .05 with Bonferroni correction, otherwise gray. (C, D, G, H) Median thickness and aCNR, respectively, overlaid onto parcellation ROIs for (C, G) 3‐T and (D, H) 9.4‐T data. Cortical thickness was estimated to be thinner at 9.4 T; the effect was mainly driven by differences in temporal lobes, insula, and cingulum. Cortex aCNR was lower at 9.4 T than at 3 T, although without correction for the reduction of voxel volume to 51% (.8 mm vs. 1.0 mm isotropic) at 9.4 T. It should be noted that we cannot exclude that noise reduction filtering in the 3‐T magnetic resonance imaging (MRI) system vendor's reconstruction pipeline might have biased this estimate in favor of the 3‐T data. Statistically significant differences were found in the lower temporal lobes, but also in central and superior temporal cortex. These areas correspond to the primary auditory, sensory, and motor cortices, which are known to be more heavily myelinated and have increased iron content. The relationship between myelination and T1 contrast invites the speculation that these findings reflect increased sensitivity of ultra‐high‐field MRI to certain features of cortical microstructure,^4^ although radiofrequency inhomogeneity effects and reconstruction artifacts could also confound them.
**TABLE S1.** Patient demographic and clinical information. F, frontal; FCD, focal cortical dysplasia; HS, hippocampal sclerosis; I, insular; L, left; O, occipital; P, parietal; R, right; T, temporal. Surgical outcome is specified as Engel class (I–IV). *Years specified in age groups (minimum 5‐year range) for deidentification.^1^ Patient P014 discontinued medication postoperatively and continued to have seizures, rendering the outcome uninterpretable. ^2^ For Patient P016, no seizures were recorded during video‐electroencephalographic monitoring, and psychogenic nonepileptic seizures were later discussed.

## Data Availability

The data that support the findings of this study are available on request from the corresponding author. The data are not publicly available due to privacy or ethical restrictions.
