# Supplementary material for: 9.4‐Tesla magnetic resonance imaging in focal epilepsy patients with high‐resolution surface‐based profiling of focal cortical dysplasias
Source: Epilepsia. 2026 Sep 2;67(11):5889–900. doi: 10.1002/epi.70418 (PMC13768927; doi:10.1002/epi.70418)
Supplement: Supplementary file 1 [file EPI-67-5889-s001.pdf]

9.4 Tesla MRI in focal epilepsy patients with high-resolution surface-based profiling of focal cortical dysplasias

Cornelius Kronlage[1,2,7], Pascal Martin[1,2], Benjamin Bender[3], Gisela E. Hagberg[4,5], Jonas Bause[4,5], Joana RA Loureiro[4,5,*], Mathilde Ripart[7,8], Sophie Adler[7,8], Konrad Wagstyl[7,8], Holger Lerche[1,2], Niels KN Focke[6], Esther Kuehn[2,9]

1 Department of Neurology and Epileptology, University of Tuebingen Medical Faculty, Tuebingen, Germany
2 Hertie Institute for Clinical Brain Research, University of Tuebingen Medical Faculty, Tuebingen, Germany
3 Department of Neuroradiology, University Hospital Tuebingen and University of Tuebingen, Tuebingen, Germany
4 Department of High-Field Magnetic Resonance, Max-Planck-Institute for Biological Cybernetics, Tuebingen, Germany
5 Biomedical Magnetic Resonance, University Hospital of Tübingen, Tübingen, Germany
6 Department of Neurology, University Medical Center Göttingen, Göttingen, Germany
7 School of Biomedical Engineering and Imaging Sciences, King's College London, London, UK
8 UCL Great Ormond Street Institute of Child Health, University College London, London, UK
9 German Center for Neurodegenerative Diseases (DZNE), Tübingen, Germany
* JRAL's current affiliation: Siemens Healthineers GmbH, Erlangen, Germany

Supplementary material

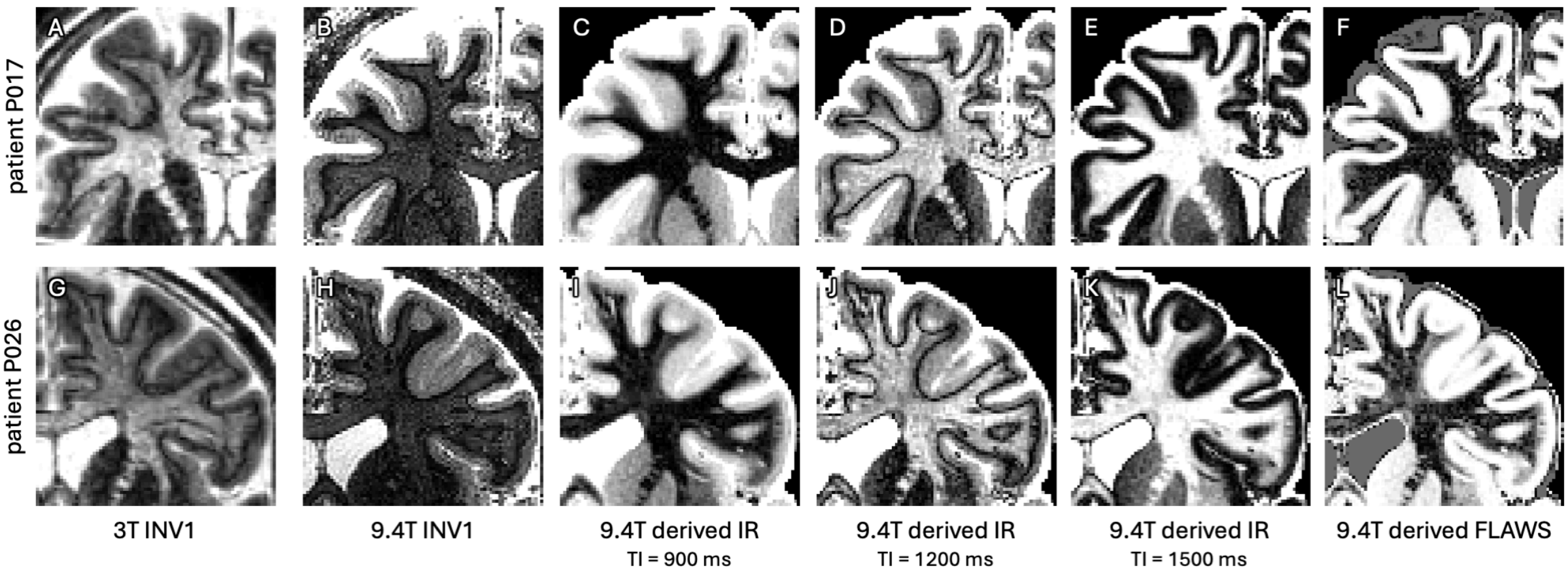

Supplementary Figure 1: Comparison of acquired and derived EDGE (edge-enhancing gradient echo) inversion recovery contrasts and FLAWS (fluid and white matter suppression) in focal cortical dysplasias. Top row (A-F) patient P017, bottom row (G-L) patient P026. (A, G): 3T MP2RAGE INV1, (B, H), 9.4T MP2RAGE INV2, (C-E and I-K) derived synthetic inversion recovery (IR) contrasts generated based on 9.4T MP2RAGE T1-map with theoretical inversion times (TI) of 900, 1200 and 1500 ms, respectively, (F, L) derived FLAWS contrast. In the 3T INV1, there is similar signal intensity in white and gray matter and a hypointense gray-white-border, similar to what has previously been termed edge-enhancing gradient echo (EDGE)[1,2]. At 9.4T, sequence parameters had not been optimized for this contrast, so that the acquired 9.4T INV1 shows hyperintense gray matter. However, it is possible to generate a derived inversion-recovery contrast in post-processing with different TIs, where a TI around 1200 ms results in an EDGE-like contrast, with smaller voxel sizes at 9.4T and better delineation of the cortex. Interestingly, EDGE-like contrasts show slight blurring and widening of the gray-white matter boundary in patient P017 (A, D). Derived fluid and white matter suppression (FLAWS[3]) images show subtle subcortical hyperintensity in patient P017 (F).

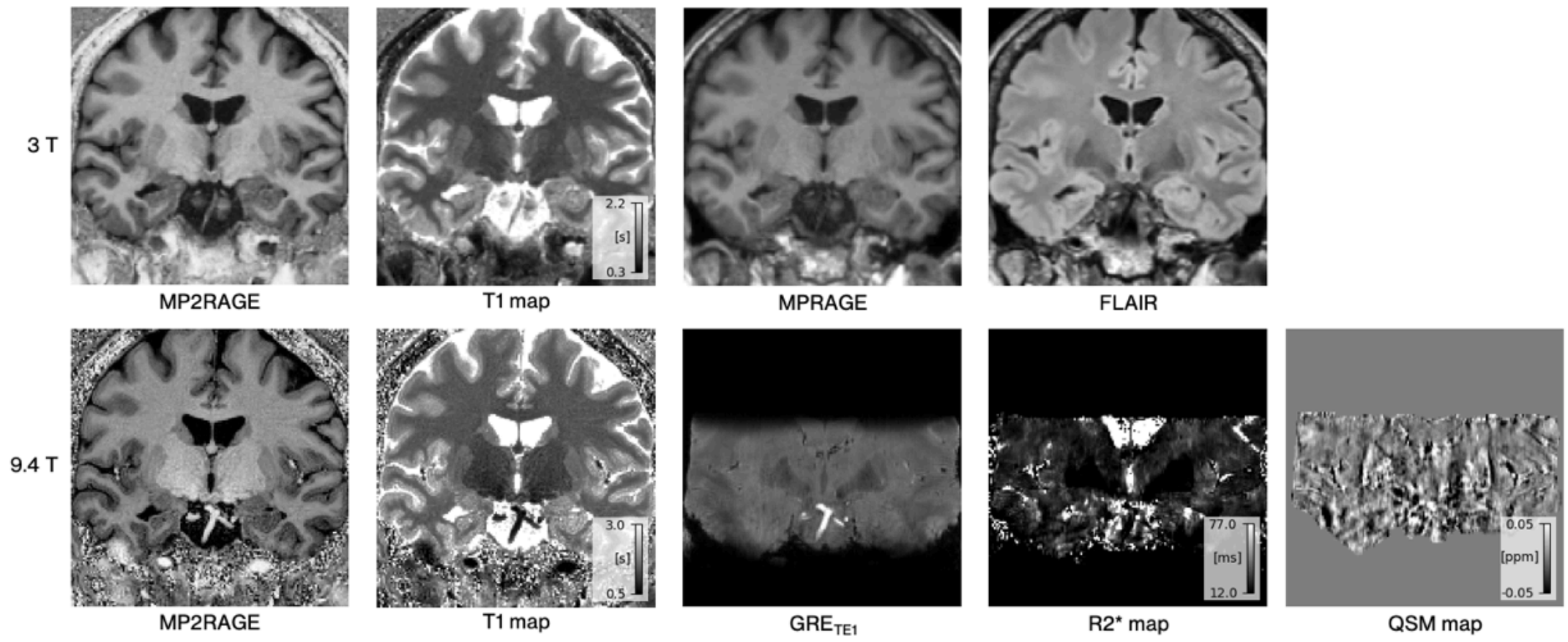

Supplementary figure 2: Coronal slices patient P009 with suspected right hippocampal sclerosis. Comparison of 3T data (top row, coregistered and resampled to the 9.4T volume with cubic interpolation) and 9.4T data (bottom row). Volume loss of the right hippocampus and corresponding enlargement of the temporal horn of the lateral ventricle can be appreciated in the 3T images. There is no clear T2/FLAIR hyperintensity. Right hippocampal volume loss is also clearly visible in the 9.4T image, with more sharply defined borders. 9.4T GRE and derived maps (T2*, QSM) do appear to show a contrast in this area and are affected by artefacts in proximity to the skull base.

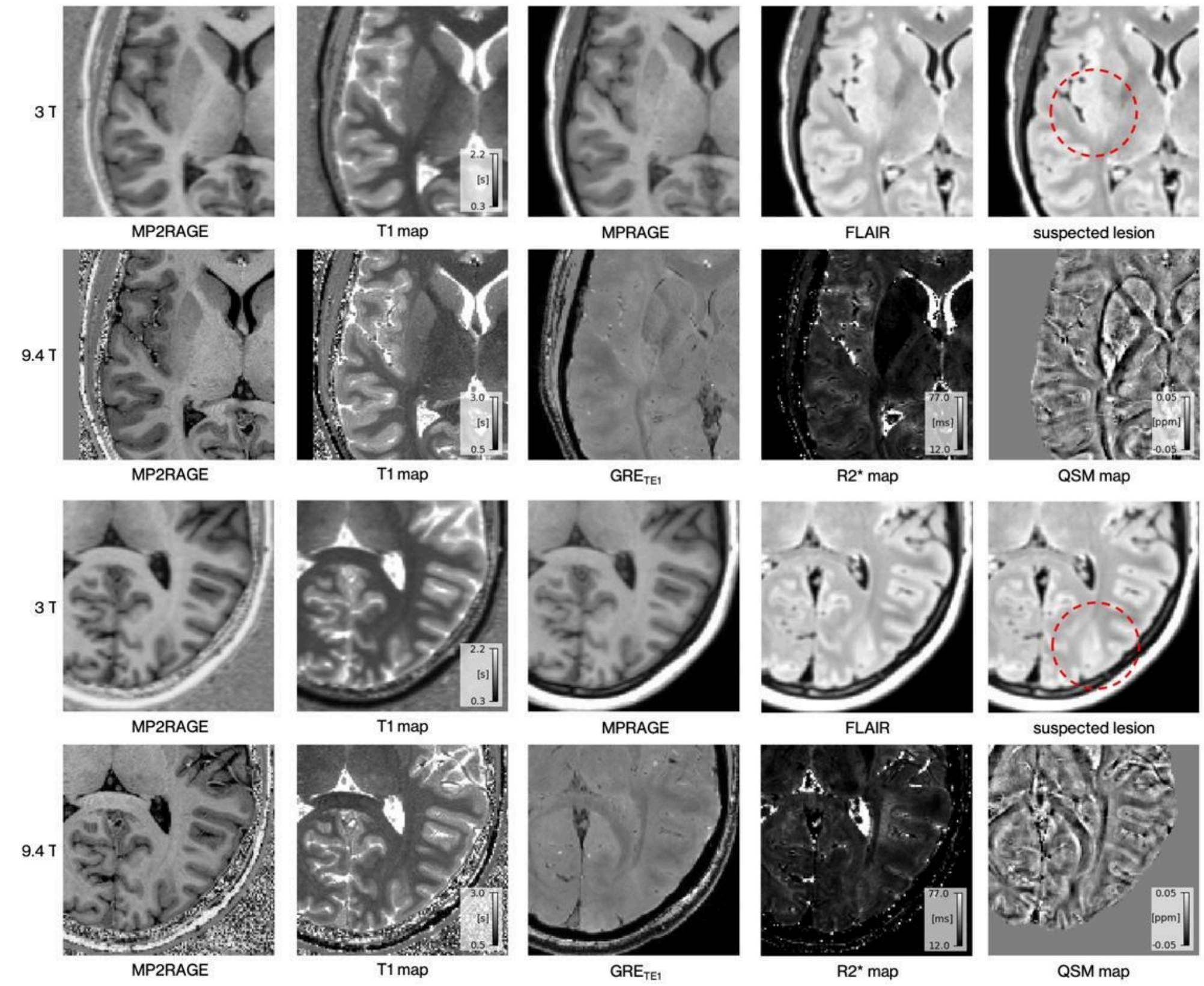

Supplementary figure 3: Axial slices of the two suspected FCD in patient P016 where no seizures could be recorded in video-EEG and a differential diagnosis of non-epileptic seizures was discussed (also rendering the diagnosis of FCDs uncertain). Top rows: An asymmetrical T2/FLAIR hyperintensity is visible in the posterior right insular cortex, in the 9.4T GRE a similar finding can be suspected, the T1-weighted sequences reveal no clear abnormality. Bottom rows: A blurring of the GM/WM boundary and subcortical FLAIR-hyperintensity is visible in the occipital sulcus in the 3T data.

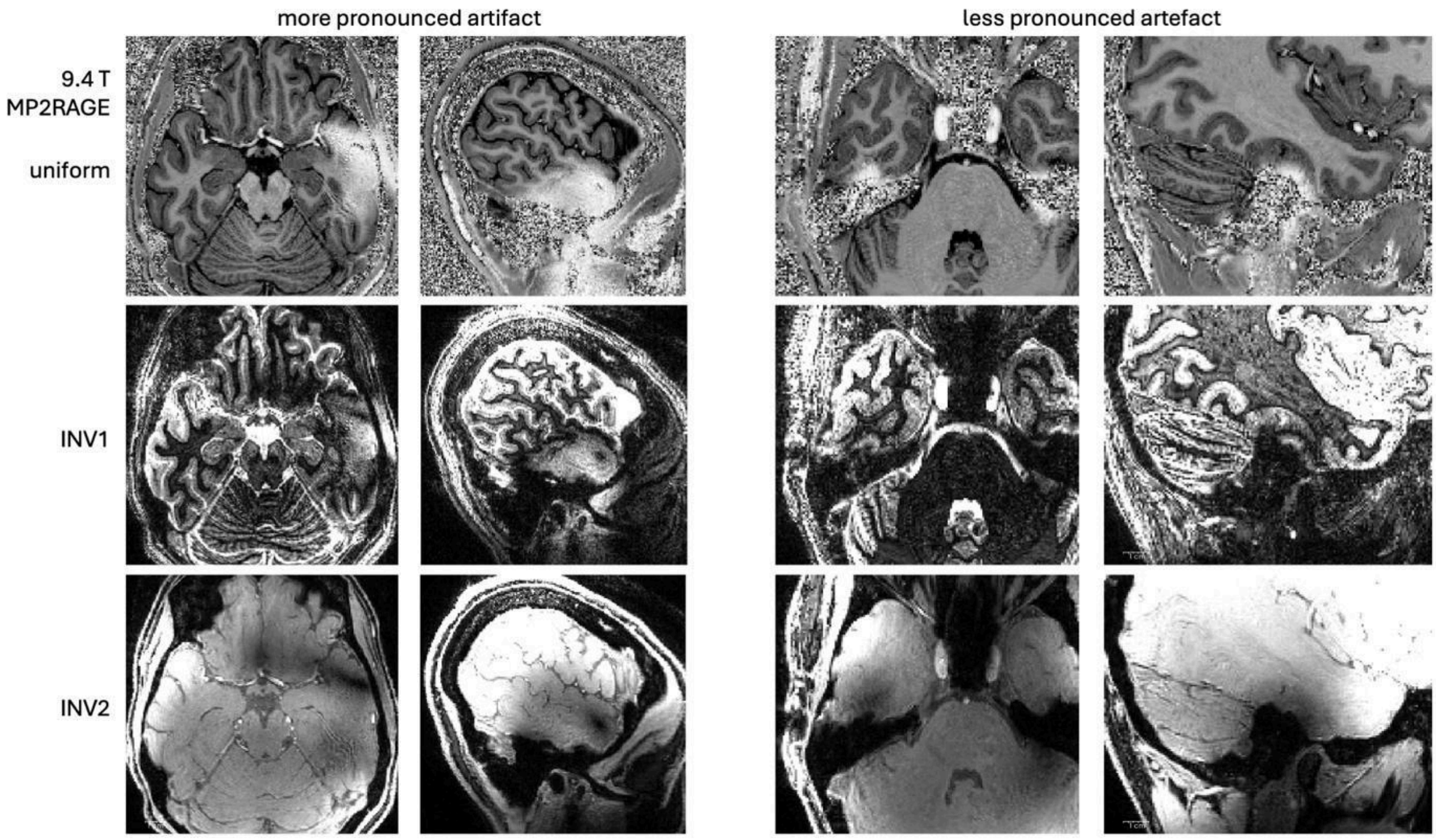

Supplementary figure 4: Artefacts in the temporal lobes in the 9.4T MP2RAGE data. Left: Example of a more pronounced artefact (as present in 3/41 subjects in total). Right: Example of a less pronounced artefact as present in all of the subjects. Top row: MP2RAGE uniform volumes, middle row: MP2RAGE INV1, bottom row: MP2RAGE INV2. These findings are likely mainly caused by transmit field inhomogeneities.

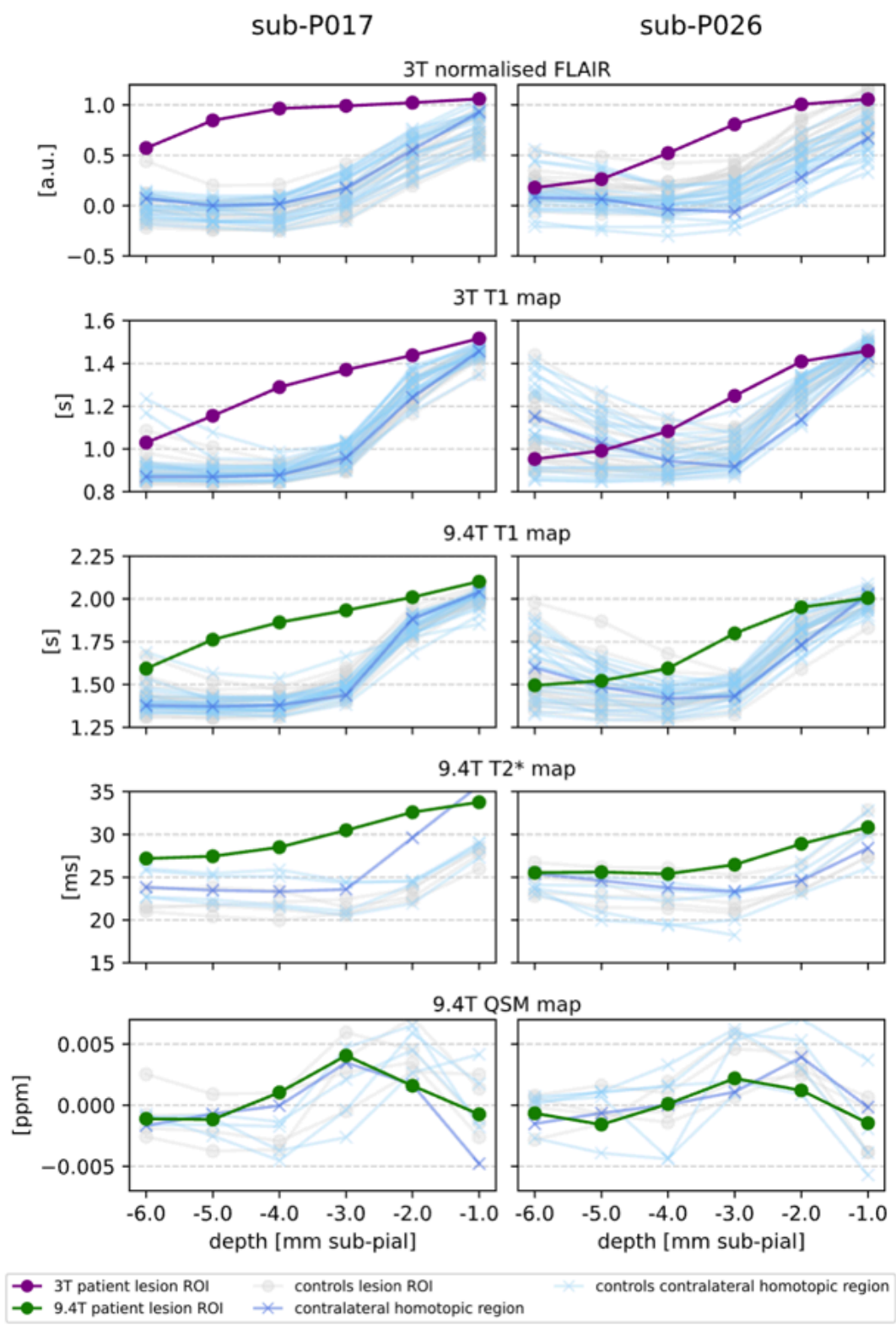

Supplementary figure 5: Vertical profiles of two histologically verified FCDs across magnetic field strengths of 3T and 9.4T. For each feature and depth, the raw feature value in the lesion ROI is plotted. Also see figure 4.

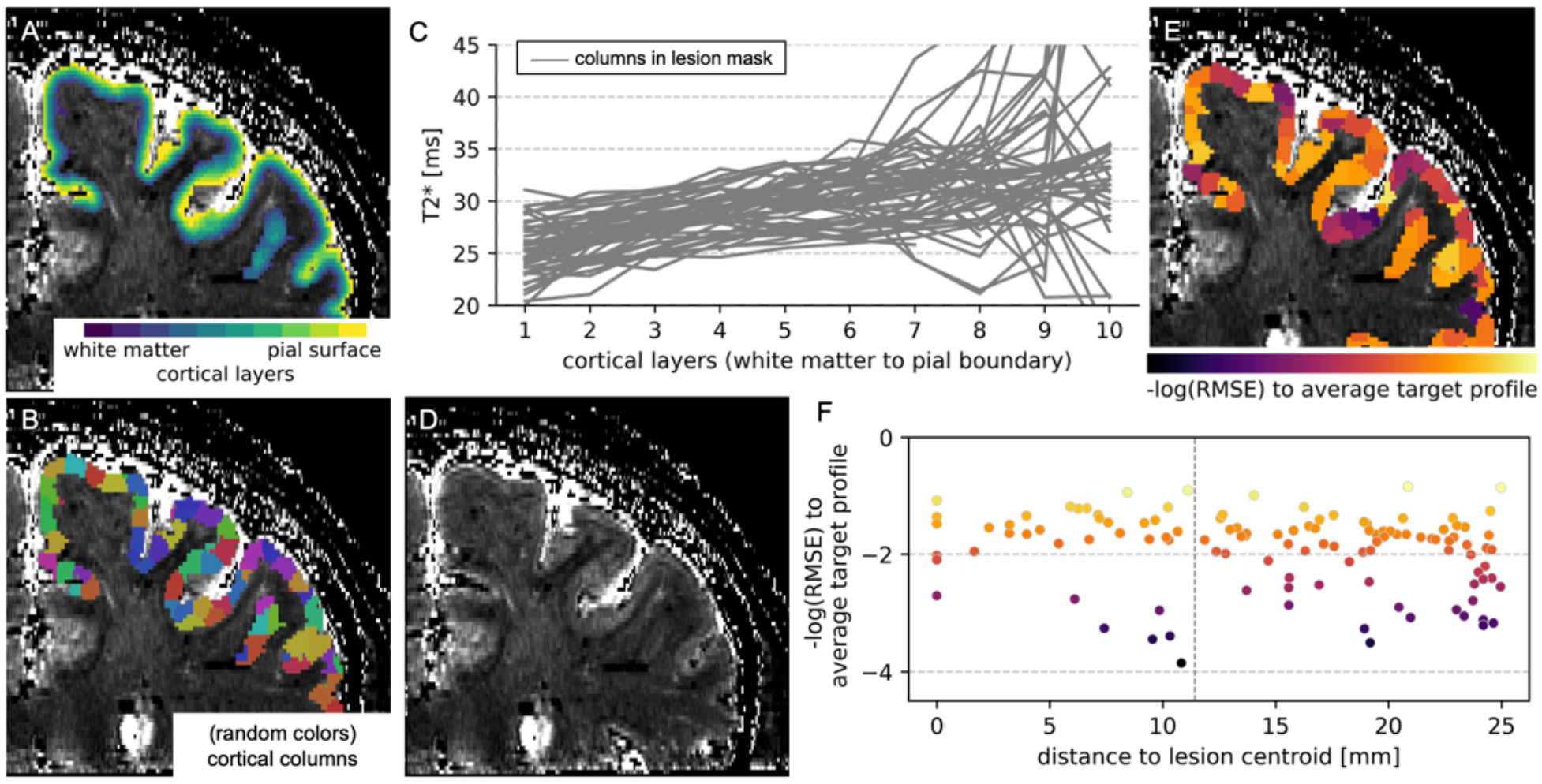

Supplementary figure 6: High resolution layer-wise analysis of T2* at 9.4T in a histologically verified FCD IIb in patient P026. Cortical voxels were assigned to 10 equivolumetric cortical layers (A) and cortical columns with a volume of 40 mm3 each (B) using the laynii software suite. Profiles of T2* vs. cortical layer were extracted, (C) shows the profiles of all columns in the manual lesion mask. In this case, there were no columns matching the manually set criteria (see methods) for the “black line sign” feature. (D) shows the T2* map without overlays. We assessed the similarity of P026 column T2* profiles to the target profile derived from patient P017. The log-transformed root mean square error (RMSE) is plotted overlaid onto the respective columns (E) and against the distance from the lesion centroid (F). In this case, although individual columns have a higher similarity score, there is no consistent distance-dependent pattern as in P017.

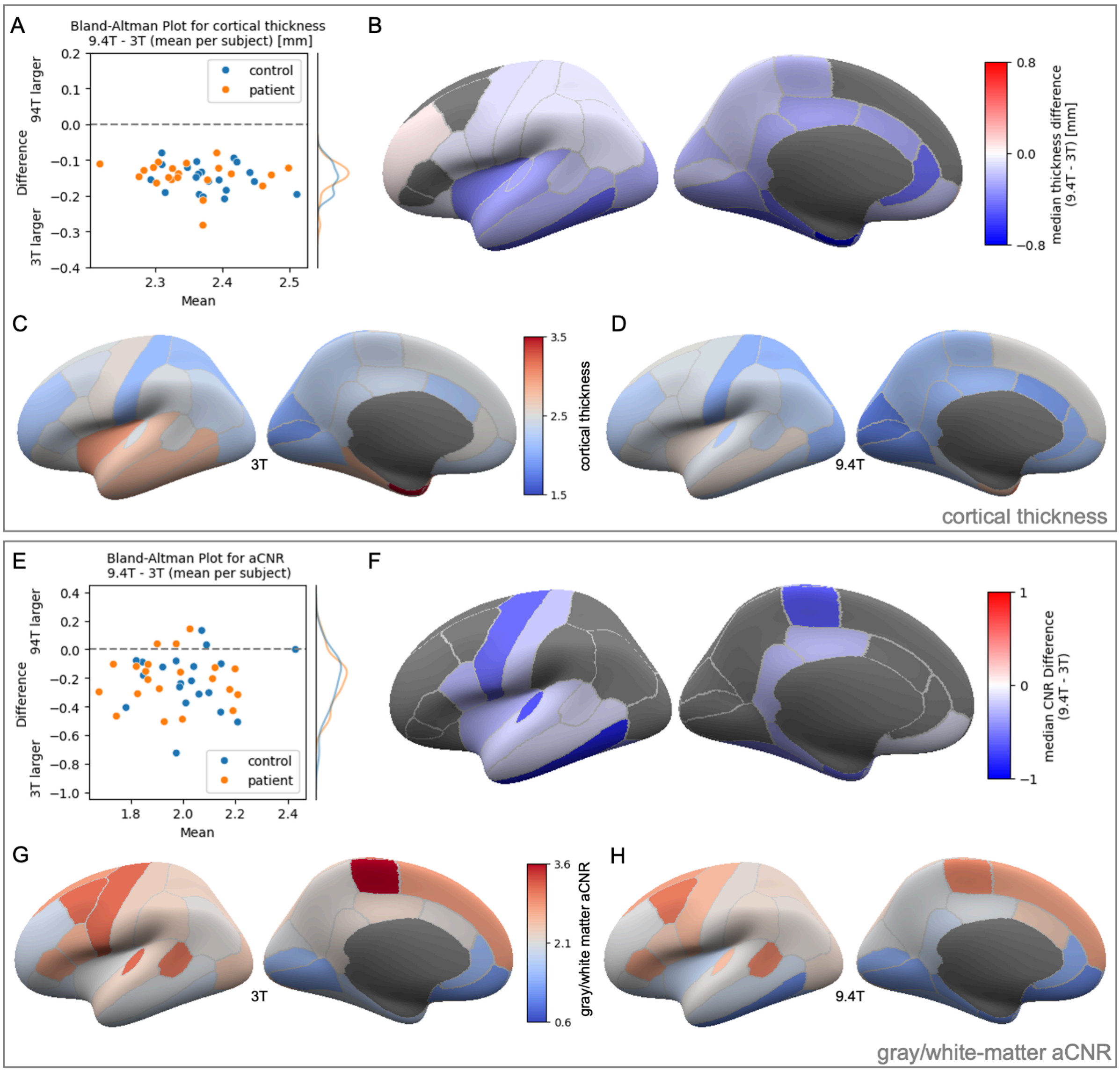

Supplementary figure 7: Comparison of cortical thickness (A-D) and white/gray-matter apparent contrast to noise ratio (aCNR) (E-H) between paired 9.4T and 3T MP2RAGE acquisitions. The plots show these data aggregated in different ways: (A, E) Bland-Altmann plots of paired 9.4T and 3T measurements averaged per subject; difference on the y-axis, mean on the x-axis, kernel density estimate plot of the difference marginal distribution on the right, colored according to group (patient vs control). For both thickness and aCNR, a mixed-effects linear regression model identified a significant effect of field strength (9.4T/3T) ($p < 0.001$), whereas group label (patient/control) and its interaction with field strength interaction were not significant. (B, F) median difference per parcellation ROI for each feature overlaid onto a template inflated surface plot, only shown if Wilcoxon test $p < 0.05$ with Bonferroni correction, otherwise grey. (C, D, G, H): median thickness and aCNR, respectively, overlaid onto parcellation ROIs for (C, G) 3T and (D, H) 9.4T data. Cortical thickness was estimated thinner at 9.4T, the effect was mainly driven by differences in temporal lobes, insula and cingulum. Cortex aCNR was lower at 9.4T than at 3T - though without correction for the reduction of voxel volume to 51% (0.8 mm vs. 1.0 mm isotropic) at 9.4T. It should be noted that we cannot exclude that noise reduction filtering in the 3T MRI system vendor's reconstruction pipeline might have biased this estimate in favor of the 3T data. Statistically significant differences were found in the lower temporal lobes - but also in central and superior temporal cortex. These areas correspond to the primary auditory, sensory, and motor cortices, which are known to be more heavily myelinated and have increased iron content.

The relationship between myelination and T1 contrast invites the speculation that these findings reflect increased sensitivity of ultra-high-field MRI to certain features of cortical microstructure[4], though RF inhomogeneity effects and reconstruction artefacts could also confound them.

id	age [years*]	sex	age at onset of epilepsy [years*]	3T MRI diagnosis	suspected pathology on 3T MRI	clinical hypothesis (lobar)	FDG PET finding/ localization	neuro-psychology	non-invasive video-EEG	invasive EEG localization	surgery performed	contraindication to surgery	histopathology	postsurgical outcome (Engel class)
P003	18-25	f	11-15	none	n/a	R F	n/a	R F-T	R F	n/a	0	n/a	n/a	n/a
P004	36-40	f	0-5	none	n/a	R F-I	normal	R F-T	R F-I	R F/I[3]	0	multifocal	n/a	n/a
P005	31-35	m	16-20	none	n/a	L T	n/a	L F-T	L T	n/a	0	n/a	n/a	n/a
P006	26-30	f	16-20	none	n/a	R T	normal	non localizing	R T	n/a	0	n/a	n/a	n/a
P008	46-50	f	31-35	none	n/a	R T	normal	L F-T	R T	n/a	0	eloquent area	n/a	n/a
P009	51-55	f	26-30	suspicion	HS R	L/R T	n/a	L/R F-T	L/R T	n/a	0	n/a	n/a	n/a
P010	41-45	m	11-15	none	n/a	L T	n/a	L F-T	L/R F-T	n/a	0	n/a	n/a	n/a
P011	18-25	m	16-20	none	n/a	L/R F	normal	L/R F	L/R F	n/a	0	n/a	n/a	n/a
P012	18-25	m	11-15	none	n/a	L T-P	normal	L F	L F-T-P	L T-P[3]	0	eloquent area	n/a	n/a
P013	26-30	f	16-20	none	n/a	L/R T	L T	L F-T	L/R T	L+R T[3]	0	multifocal	n/a	n/a
P014	31-35	f	0-5	suspicion	FCD	L C	L F	L F-T	L F	L F	1	n/a	fragmented tissue, no FCD	not classifiable[1]
P015	18-25	f	11-15	none	n/a	L/R F	normal	L/R F	L/R F	n/a	0	n/a	n/a	n/a
P016	26-30	f	6-10	suspicion	FCD	R I, L O	n/a	F	no seizures recorded[2]	n/a	0	n/a	n/a	n/a
P017	18-25	f	0-5	positive	FCD	R F	n/a	F	R F	n/a	1	n/a	FCD IIb	I (6 years)
P018	26-30	m	11-15	none	n/a	L F	normal	L/R F	L F	n/a	0	n/a	n/a	n/a
P021	18-25	f	11-15	none	n/a	L F	n/a	L/R F	L F	n/a	0	n/a	n/a	n/a
P022	18-25	m	6-10	none	n/a	R T-P-O	normal	R T-P	R T-P-O	R T-P-O	0	eloquent area	n/a	n/a
P023	18-25	m	0-5	none	n/a	R F-C-T	normal	R F-T	R F-T	n/a	0	n/a	n/a	n/a
P024	31-35	m	16-20	none	n/a	R T	normal	R F-T	R F-T	n/a	0	n/a	n/a	n/a
P025	26-30	m	11-15	none	n/a	L/R T	normal	R F-T	L/R T	n/a	0	n/a	n/a	n/a
P026	46-50	m	31-35	positive	FCD	L F	n/a	L F-T	L F	L F	1	n/a	FCD IIb	I (min. 2 years)

Supplementary Table 1: Patient demographic and clinical information, L: left, R: right, F: frontal, I: insular, T: temporal, P: parietal, O: occipital, HS: hippocampal sclerosis, FCD: focal cortical dysplasia. Surgical outcome specified as Engel class (I-IV). *years specified in age groups (min. 5 years range) for de-identification. Footnotes: (1) Patient P014 discontinued medication postoperatively and continued to have seizures, rendering the outcome uninterpretable. (2) For patient P016, no seizures were recorded during video-EEG monitoring, and psychogenic non-epileptic seizures were later discussed.

Supplementary Methods: Surface-based analysis

1. Segmentation and cortical surface reconstruction

MP2RAGE ‘uniform’ T1-weighted images contain salt and pepper noise outside the brain that impedes processing with pipelines adapted to conventional T1-weighted data such as Freesurfer. Therefore, 9.4T / 3T MP2RAGE images were denoised by calculating ‘robust’ uniform T1w images based on the GRE_{TI1} and GRE_{TI2} data[5] with the parameter beta set to 0.4. As the denoising reintroduces some bias field artifacts, N4 bias field correction[6] was applied subsequently.

The denoised uniform 9.4T and 3T MP2RAGE images, respectively, were then used for segmentation and surface reconstruction using the Freesurfer recon-all pipeline,[7] version 7.4.1, set to operate at native sub-millimetric resolution (‘-hires’ flag) and to use synthstrip[8] for improved skull stripping quality.

2. Surface-based features and lesion profiles

The 3T MPRAGE T1w and FLAIR volumes were coregistered (using Freesurfer’s ‘mri_coreg’ utility with 12 degrees of freedom), resampled to the uniform 3T MP2RAGE volumes, N4 bias field corrected, and intensity normalised (by linearly rescaling so that the medians of white matter and cortex voxels are 1 and 0, respectively – similar to a method described previously for FLAIR[9]).

The 9.4T GRE_{TE1} volumes were coregistered to the 9.4T MP2RAGE volumes (using mri_coreg as well). This registration was used to align and resample T2*- and QSM-maps accordingly.

All available volume data (at 3T: uniform MP2RAGE, T1-maps, normalised MPRAGE, normalised FLAIR; at 9.4T: uniform MP2RAGE, T1-maps, T2*-maps, QSM-maps) were projected onto the respective cortical surface mesh at six depths ranging from –1.0 to –6.0 mm from the pial surface in 1.0 mm increments. Cortical thickness was re-estimated without the Freesurfer default upper limit of 5.0 mm.

3. Registration to a template space

Surface meshes were coregistered to a bilaterally symmetric template (xhemi, fsaverage_sym)[10] to enable per-vertex comparisons between subjects. All surface features were mapped to this template space.

Similarly, lesion masks were sampled onto the respective subject’s cortical surface mesh and then to the Freesurfer template. To avoid a bias towards one field strength, the set of template vertices identified as lesional based on both 3T and 9.4T reconstructions (i.e., the intersection) was used for further analysis. Similarly, the per-vertex geodesic distance to the lesion centroid (lesional vertex with the smallest distance to the center of mass of all lesional vertices) was computed along the pial surface for both 3T and 9.4T surface meshes and averaged on the template. The lesion masks in the left-right symmetric template space also defined homotopic regions on the contralateral hemisphere for patients and in controls.

4. Analysis and statistics

Surface-based features were compared between 9.4T and 3T for the n=2 histologically verified FCDs. Vertex-wise data was aggregated by computing the median either in the entire lesion mask (for comparisons across cortical depth) or in bins with equal numbers of vertices in increasing geodesic distance from the lesion mask centroid. Features were normalized by z-scoring (subtraction of mean and division by standard deviation) with respect to the distribution of healthy control subjects. Due to the limited sample size of histopathologically confirmed FCDs (n=2), we did not compute statistical comparisons on this data.

5. Comparison of surface-based image features across field strengths

Cortical thickness and cortical apparent contrast to noise ratio (aCNR) were selected as key image features relevant to detection of cortical epileptogenic lesions.
For estimating aCNR, we sampled the image intensities at depth 50% cortical thickness (for grey matter) and 2 mm subcortically (for white matter) and defined

$$aCNR_{gray\text{-}white} = |(mean_{gray} - mean_{white}) / sqrt(SD^2_{gray} + SD^2_{white})|$$

similar to other radiological literature[11] because this definition does not depend on noise estimation in background air regions, which would not be meaningful in MP2RAGE. For analysis, we aggregated these features in two ways: Firstly, by averaging across the whole cortex per subject. This data is shown in Bland-Altmann plots (difference vs. mean of 9.4T vs 3T estimates). We computed a mixed-effects linear regression model with field strength (9.4T/3T), group (patient/control) and their interaction as categorical fixed effects and subject as random effect. Secondly, by averaging across all subjects in ROIs according to the Desikan-Killiany cortical parcellation. Results are plotted overlaid on the template (fsaverage_sym) inflated cortical surface. To compare 9.4T and 3T data, we calculated the median paired difference and computed a Wilcoxon signed-rank test with Bonferroni correction for parcellation ROIs.

Supplementary Discussion: 9.4T MRI acquisitions and sequence design

Parallel transmit methodology (pTx) can reduce RF inhomogeneity and lower SAR at the same time. It has been shown to improve diagnostic performance in focal epilepsies at 7T[12]. When measurements for this study were performed (between 2015 and 2018), it was not yet possible to apply pTx pulses robustly at 9.4T. The development of pTx universal pulses, which has now been successfully applied for 9.4T MRI[13], is likely to improve image homogeneity in future studies, and enable MRI with T2-FLAIR. In the recent case report of an epilepsy patient examined at 9.4T by van Lanen and coworkers using custom pTx universal pulses, RF inhomogeneity artefacts, especially in the temporal lobes, were still present in MPRAGE, and T2-weighted sequences were not acquired due to SAR limitations[14]. The improvement of MR image quality based on pTx technology is therefore still subject to methodological research and development.

The sequence design for this study was based on previous measurements conducted at the 9.4T MRI system and pilot measurements. Requirements included achieving both high spatial resolution and SNR, quantitative imaging capability, whole brain coverage, compensation for RF inhomogeneity, meeting SAR safety limits and a feasible acquisition time. The MP2RAGE sequence meets these criteria and is a common choice for UHF MRI[15,16]. We show that MP2RAGE-based qT1 estimates can be used to generate derived EDGE and FLAWS contrasts (Supplementary Figure 1), which have been shown to enhance visibility of FCDs[1–3]. Generally, trade-offs between some parameters have to be weighed; SNR is proportional to the voxel volume (i.e. voxel side length cubed) and the square root of acquisition time. For future 9.4T MRI acquisitions, methodological advances that were made meanwhile would allow better image quality beyond these considerations, e.g., accelerated imaging, CAIPIRINHA undersampling, compressed sensing, deep-learning based image reconstruction[17], FLAIR sequences acquired at ultra-high field MRI, and acquisition of T2* contrast with EPI[18].

References

1. Middlebrooks EH, Lin C, Westerhold E, Okromelidze L, Vibhute P, Grewal SS, et al. Improved detection of focal cortical dysplasia using a novel 3D imaging sequence: Edge-Enhancing Gradient Echo (3D-EDGE) MRI. NeuroImage: Clinical. 2020; 28:102449.

2. Middlebrooks EH, Greco E, Zhou X, Gupta V, Freund BE, Agarwal AK, et al. Edge-Enhancing Gradient Echo MRI at 7T for detection of focal cortical dysplasia in epilepsy. Neuroimage: Reports. 2023; 3(4):100187.

3. Chen X, Qian T, Kober T, Zhang G, Ren Z, Yu T, et al. Gray-matter-specific MR imaging improves the detection of epileptogenic zones in focal cortical dysplasia: A new sequence called fluid and white matter suppression (FLAWS). NeuroImage: Clinical. 2018; 20:388–97.

4. Glasser MF, Van Essen DC. Mapping Human Cortical Areas In Vivo Based on Myelin Content as Revealed by T1- and T2-Weighted MRI. Journal of Neuroscience. 2011; 31(32):11597–616.

5. O'Brien KR, Kober T, Hagmann P, Maeder P, Marques J, Lazeyras F, et al. Robust T1-Weighted Structural Brain Imaging and Morphometry at 7T Using MP2RAGE. PLOS ONE. 2014; 9(6):e99676.

6. Tustison NJ, Avants BB, Cook PA, Zheng Y, Egan A, Yushkevich PA, et al. N4ITK: Improved N3 Bias Correction. IEEE Transactions on Medical Imaging. 2010; 29(6):1310–20.

7. Fischl B. FreeSurfer. NeuroImage. 2012; 62(2):774–81.

8. Hoopes A, Mora JS, Dalca AV, Fischl B, Hoffmann M. SynthStrip: skull-stripping for any brain image. NeuroImage. 2022; 260:119474.

9. Focke NK, Symms MR, Burdett JL, Duncan JS. Voxel-based analysis of whole brain FLAIR at 3T detects focal cortical dysplasia. Epilepsia. 2008; 49(5):786–93.

10. Greve DN, Van der Haegen L, Cai Q, Stufflebeam S, Sabuncu MR, Fischl B, et al. A Surface-based Analysis of Language Lateralization and Cortical Asymmetry. Journal of Cognitive Neuroscience. 2013; 25(9):1477–92.

11. Fervers P, Zaeske C, Rauen P, Iuga A-I, Kottlors J, Persigehl T, et al. Conventional and Deep-Learning-Based Image Reconstructions of Undersampled K-Space Data of the Lumbar Spine Using Compressed Sensing in MRI: A Comparative Study on 20 Subjects. Diagnostics. 2023; 13(3):418.

12. Klodowski K, Zhang M, Jen JP, Scoffings DJ, Morris R, Lupson V, et al. Parallel transmit 7T MRI for adult epilepsy pre‐surgical evaluation. Epilepsia. 2025; 66(7):2315–27.

13. Wiggins C, Poser B, Mauconduit F, Boulant N, Gras V. Universal Pulses for MRI at 9.4 Tesla - a Feasibility Study. In: 2019 International Conference on Electromagnetics in Advanced Applications (ICEAA) [Internet]. 2019 [cited 2026]. p. 1185–8. Available from: https://ieeexplore.ieee.org/document/8879180/

14. Van Lanen RHGJ, Uher D, Tse DHY, Steijvers E, Colon AJ, Jansen JFA, et al. In vivo 9.4 Tesla MRI of a patient with drug-resistant epilepsy: Technical report. Acta Neurochir. 2025; 167(1):18.

15. van Lanen RHGJ, Colon AJ, Wiggins CJ, Hoeberigs MC, Hoogland G, Roebroeck A, et al. Ultra-high field magnetic resonance imaging in human epilepsy: A systematic review. NeuroImage: Clinical. 2021; 30:102602.

16. Marques JP, Kober T, Krueger G, van der Zwaag W, Van de Moortele P-F, Gruetter R. MP2RAGE, a self bias-field corrected sequence for improved segmentation and T1-mapping at high field. NeuroImage. 2010; 49(2):1271–81.

17. Middlebrooks EH, Ekert JO, Zhou X, Tao S, Patel VN, Yu T, et al. A Comparative Evaluation of 7T MRI for Epilepsy with Deep-Learning–Based Image Reconstruction and Dynamic Parallel Transmission. AJNR Am J Neuroradiol. 2026; :ajnr.A9218.

18. Stirnberg R, Deistung A, Reichenbach JR, Breteler MMB, Stöcker T. Rapid submillimeter QSM and R2* mapping using interleaved multishot 3D-EPI at 7 and 3 Tesla. Magnetic Resonance in Medicine. 2024; 92(6):2294–311.
